# Meta-analysis of Immunochip data of four autoimmune diseases reveals novel single-disease and cross-phenotype associations

**DOI:** 10.1186/s13073-018-0604-8

**Published:** 2018-12-20

**Authors:** Ana Márquez, Martin Kerick, Alexandra Zhernakova, Javier Gutierrez-Achury, Wei-Min Chen, Suna Onengut-Gumuscu, Isidoro González-Álvaro, Luis Rodriguez-Rodriguez, Raquel Rios-Fernández, Miguel A. González-Gay, Coeliac Disease Immunochip Consortium, Gosia Trynka, Karen A. Hunt, Nicholas A. Bockett, Jihane Romanos, Vanisha Mistry, Agata Szperl, Sjoerd F. Bakker, Maria Teresa Bardella, Leena Bhaw-Rosun, Gemma Castillejo, Emilio G. de la Concha, Rodrigo Coutinho de Almeida, Kerith-Rae M. Dias, Cleo C. van Diemen, Patrick C. A. Dubois, Richard H. Duerr, Sarah Edkins, Lude Franke, Karin Fransen, Javier Gutierrez, Graham A. R. Heap, Barbara Hrdlickova, Sarah Hunt, Leticia Plaza Izurieta, Valentina Izzo, Leo A. B. Joosten, Cordelia Langford, Maria Cristina Mazzilli, Charles A. Mein, Vandana Midah, Mitja Mitrovic, Barbara Mora, Marinita Morelli, Sarah Nutlan, Kerra Pearce, Mathieu Platteel, Isabel Polanco, Simon Potter, Carmen Ribes-Koninckx, Isis Ricaño-Ponce, Anna Rybak, José Luis Santiago, Sabyasachi Senapati, Ajit Sood, Hania Szajewska, Riccardo Troncone, Jezabel Varadé, Chris Wallace, Victorien M. Wolters, Alexandra Zhernakova, B. K. Thelma, Bozena Cukrowska, Elena Urcelay, Jose Ramon Bilbao, M. Luisa Mearin, Donatella Barisani, Jeffrey C. Barrett, Vincent Plagnol, Cisca Wijmenga, David A. van Heel, Rheumatoid Arthritis Consortium International for Immunochip (RACI), Steve Eyre, John Bowes, Dorothée Diogo, Annette Lee, Anne Barton, Paul Martin, Alexandra Zhernakova, Eli Stahl, Sebastien Viatte, Kate McAllister, Christopher I. Amos, Leonid Padyukov, Rene E. M. Toes, Tom W. J. Huizinga, Cisca Wijmenga, Gosia Trynka, Lude Franke, Harm-Jan Westra, Lars Alfredsson, Xinli Hu, Cynthia Sandor, Sonia Davila, Chiea Chuen Khor, Khai Koon Heng, Robert Andrews, Sarah Edkins, Sarah E. Hunt, Cordelia Langford, Deborah Symmons, Pat Concannon, Stephen S. Rich, Miguel A. Gonzalez-Gay, Luis Rodriguez-Rodriguez, Lisbeth Ärlsetig, Javier Martin, Solbritt Rantapää-Dahlqvist, Robert M. Plenge, Soumya Raychaudhuri, Lars Klareskog, Peter K. Gregersen, Jane Worthington, International Scleroderma Group, Maureen D. Mayes, Lara Bossini-Castillo, Olga Gorlova, Xiaodong Zhou, Wei V. Chen, Shervin Assassi, Jun Ying, Filemon K. Tan, Frank C. Arnett, John D. Reveille, Sandra Guerra, Peter K. Gregersen, Annette T. Lee, Carmen Pilar Simeón, Patricia Carreira, Iván Castellví, Miguel A. González-Gay, Lorenzo Beretta, Alexander E. Voskuyl, Paolo Airò, Claudio Lunardi, Paul Shiels, Jacob M. van Laar, Ariane Herrick, Jane Worthington, Christopher P. Denton, Jasper Broen, Timothy R. D. J. Radstake, Carmen Fonseca, Bobby P. Koeleman, Javier Martin, Raquel Ríos, Jose Luis Callejas, José Antonio Vargas Hitos, Rosa García Portales, María Teresa Camps, Antonio Fernández-Nebro, María F. González-Escribano, Francisco José García-Hernández, Mª. Jesús Castillo, Mª. Ángeles Aguirre, Inmaculada Gómez-Gracia, Luis Rodríguez-Rodríguez, Paloma García de la Peña, Esther Vicente, José Luis Andreu, Mónica Fernández de Castro, Francisco Javier López-Longo, Lina Martínez, Vicente Fonollosa, Alfredo Guillén, Gerard Espinosa, Carlos Tolosa, Anna Pros, Mónica Rodríguez Carballeira, Francisco Javier Narváez, Manel Rubio Rivas, Vera Ortiz-Santamaría, Ana Belén Madroñero, Bernardino Díaz, Luis Trapiella, Adrián Sousa, María Victoria Egurbide, Patricia Fanlo Mateo, Luis Sáez-Comet, Federico Díaz, Vanesa Hernández, Emma Beltrán, José Andrés Román-Ivorra, Elena Grau, Juan José Alegre-Sancho, Francisco J. Blanco García, Natividad Oreiro, Norberto Ortego-Centeno, Mayka Freire, Benjamín Fernández-Gutiérrez, Alejandro Balsa, Ana M. Ortiz, Jeska de Vries-Bouwstra, Cesar Magro-Checa, Alessandro Santaniello, Chiara Bellocchi, Gianluca Moroncini, Armando Gabrielli, Type 1 Diabetes Genetics Consortium, Ellen Adlem, James Allen, Jeffrey Barrett, Judy Brown, Oliver Burren, Pamela Clarke, David Clayton, Gillian Coleman, Jason Cooper, Francesco Cucca, Lucy Davison, Kate Downes, Simon Duley, David Dunger, Laura Esposito, Vin Everett, Sarah Field, Jason Hafler, Matthew Hardy, Deborah Harrison, Inge Harrison, Steve Hawkins, Barry Healy, Simon Hood, Simon Howell, Meeta Maisuria, William Meadows, Trupti Mistry, Sergey Nezhenstsev, Sarah Nutland, Nigel Ovington, Vincent Plagnol, Dan Rainbow, Kara Rainbow, Srilakshmi Raj, Helen Schuilenburg, Anna Simpson, Luc Smink, Debbie Smyth, Helen Stevens, Niall Taylor, John Todd, Jaakko Tuomilehto, Neil Walker, Linda Wicker, Barry Widmer, Mark Wilson, Heather Withers, Jennie Yang, Mark Brown, Arnetta Crews, Jason Griffin, Mark Hall, Teresa Harnish, John Hepler, Joan Hilner, Nancy King, Kurt Lohman, Lingyi Lu, Josyf Mychaleckyj, Jay Nail, Letitia Perdue, June Pierce, David Reboussin, Scott Rushing, Michele Sale, Elizabeth Sides, Beverly Snively, Hoa Teuschler, Goodrich Theil, Lynne Wagenknecht, Dustin Williams, Maureen D. Mayes, Soumya Raychaudhuri, Stephen S. Rich, Cisca Wijmenga, Javier Martín

**Affiliations:** 1 Instituto de Parasitología y Biomedicina “López-Neyra”, CSIC, PTS Granada, Granada, Spain; 2 Systemic Autoimmune Disease Unit, Instituto de Investigación Biosanitaria de Granada, Granada, Spain; 3 Department of Genetics, University of Groningen, University Medical Centre Groningen, Groningen, The Netherlands; 4 Wellcome Trust Sanger Institute, Wellcome Trust Genome Campus, Hinxton, UK; 5 Center for Public Health Genomics, University of Virginia, Charlottesville, VA USA; 6 Rheumatology Service, Hospital Universitario La Princesa, IIS-IP, Madrid, Spain; 7 Rheumatology Service, Hospital Clinico San Carlos, IdiSSC, Madrid, Spain; 8 Systemic Autoimmune Diseases Unit, Complejo Hospitalario de Granada, Hospital Campus de la Salud, Granada, Spain; 9 Epidemiology, Genetics and Atherosclerosis Research Group on Systemic Inflammatory Diseases, IDIVAL, Santander, Spain; 10 Division of Rheumatology and Clinical Immunogenetics, The University of Texas Health Science Center-Houston, Houston, USA; 11 Division of Rheumatology, Immunology, and Allergy, Brigham and Women’s Hospital, Harvard Medical School, Boston, MA USA; 12 Division of Genetics, Brigham and Women’s Hospital, Harvard Medical School, Boston, MA USA; 13 Program in Medical and Population Genetics, Broad Institute of MIT and Harvard, Cambridge, MA USA

**Keywords:** Celiac disease, Rheumatoid arthritis, Systemic sclerosis, Type 1 diabetes, Cross-disease meta-analysis, Immunochip, Autoimmune disease, functional enrichment analysis

## Abstract

**Background:**

In recent years, research has consistently proven the occurrence of genetic overlap across autoimmune diseases, which supports the existence of common pathogenic mechanisms in autoimmunity. The objective of this study was to further investigate this shared genetic component.

**Methods:**

For this purpose, we performed a cross-disease meta-analysis of Immunochip data from 37,159 patients diagnosed with a seropositive autoimmune disease (11,489 celiac disease (CeD), 15,523 rheumatoid arthritis (RA), 3477 systemic sclerosis (SSc), and 6670 type 1 diabetes (T1D)) and 22,308 healthy controls of European origin using the R package ASSET.

**Results:**

We identified 38 risk variants shared by at least two of the conditions analyzed, five of which represent new pleiotropic *loci* in autoimmunity. We also identified six novel genome-wide associations for the diseases studied. Cell-specific functional annotations and biological pathway enrichment analyses suggested that pleiotropic variants may act by deregulating gene expression in different subsets of T cells, especially Th17 and regulatory T cells. Finally, drug repositioning analysis evidenced several drugs that could represent promising candidates for CeD, RA, SSc, and T1D treatment.

**Conclusions:**

In this study, we have been able to advance in the knowledge of the genetic overlap existing in autoimmunity, thus shedding light on common molecular mechanisms of disease and suggesting novel drug targets that could be explored for the treatment of the autoimmune diseases studied.

**Electronic supplementary material:**

The online version of this article (10.1186/s13073-018-0604-8) contains supplementary material, which is available to authorized users.

## Background

Autoimmune diseases present a complex etiology resulting from the interaction between both genetics and environmental factors. Although these conditions differ in their clinical manifestations, the existence of familial clustering across them as well as the co-occurrence of multiple immune-mediated disorders in the same individual points to the existence of a common genetic background in autoimmunity [1].

As a matter of fact, genomic studies have revealed that many genetic *loci* are associated with multiple immune-mediated phenotypes, thus suggesting that autoimmune disorders are likely to share molecular mechanisms of disease pathogenesis [2, 3]. In the last years, several approaches have been conducted to comprehensively explore this genetic overlap. In this regard, combined analysis of GWAS (genome-wide association study) or Immunochip data across multiple diseases simultaneously has emerged as a powerful strategy to identify novel pleiotropic risk *loci* as well as common pathogenic mechanisms in autoimmunity [4, 5]. Recently, a cross-phenotype study combining Immunochip data from five seronegative autoimmune diseases, including ankylosing spondylitis, Crohn’s disease (CD), psoriasis, primary sclerosing cholangitis and ulcerative colitis, identified numerous multidisease signals, some of which represented new pleiotropic risk *loci* in autoimmunity [4].

Considering the above, we decided to perform a similar approach by exploring genetic overlap across four seropositive autoimmune diseases. Specifically, Immunochip data from 37,159 patients with celiac disease (CeD), rheumatoid arthritis (RA), systemic sclerosis (SSc) and type 1 diabetes (T1D) and 22,308 unaffected individuals were combined in a cross-disease meta-analysis. The aims of this study were (i) to identify new susceptibility *loci* shared by subsets of these four immune-related conditions, (ii) to identify new associations for individual diseases, and (iii) to shed light into the molecular mechanisms shared among these four disorders by integrating genotype and functional annotation data.

## Methods

### Study population

All samples were genotyped using Immunochip (Illumina, Inc., CA), a custom array designed for dense genotyping of 186 established genome-wide significant *loci*. The cohorts included in the present study are described in Additional file 1: Table S1. The CeD cohort, composed of 11,489 cases from Italy, the Netherlands, Spain, and the UK, and the RA cohort, which included 13,819 cases from Spain, the Netherlands, Sweden, the UK, and the USA, came from a previous published meta-Immunochip [6]. In addition, 1788 RA samples from Spain (which did not overlap with the Spanish RA cases included in the Immunochip mentioned) were also analyzed. These patients were recruited in three different Spanish hospitals (Hospital Marqués de Valdecilla, Santander, Hospital Clínico San Carlos, Madrid and Hospital La Princesa, Madrid) and were diagnosed with RA according to the 1987 classification criteria of the American College of Rheumatology [7]. The T1D set consisted of 6670 cases from the UK and has been described in a previous Immunochip study [8]. Finally, the SSc cohort, which consisted of 3597 cases from Spain, the USA, the UK, Italy, and the Netherlands, was also described in a previous Immunochip study [9].

Additionally, 22,365 ethnically matched control individuals were analyzed. As indicated in Additional file 1: Table S1, some of the control sets, specifically those from Italy, the Netherlands, Spain, and the UK, overlapped among different diseases, which was taken into account for the subsequent cross-disease meta-analysis.

### Quality control and imputation

Before imputation, data quality control was performed separately for each cohort using PLINK 1.9 [10]. Single-nucleotide polymorphisms (SNPs) with low call rates (< 98%), low minor allele frequency (MAF < 0.01) and those that were not in Hardy-Weinberg equilibrium (HWE; *p* < 0.001) were excluded. Individuals with successful call rates lower than 95% were also removed. Additionally, an individual of each pair of duplicates and first-degree relatives identified via the Genome function in PLINK 1.9 (PI-HAT > 0.4) was randomly discarded.

IMPUTE V.2 was used to perform SNP genotype imputation [11] using the 1000 Genomes Phase III as reference panel [12]. To maximize the quality of imputed SNPs, a probability threshold for merging genotypes of 0.9 was established. Imputation accuracy, measured as the correlation between imputed and true genotypes, considering the best-guess imputed genotypes (> 0.9 probability) was higher than 99% for all the analyzed cohorts. Imputed data were subsequently subjected to stringent quality filters in PLINK 1.9. Again, we filtered out SNPs with low call rates (< 98%) and low MAF (< 0.01) and those that deviated from HWE (*p* < 0.001). Moreover, after merging case/control sets, singleton SNPs and those showing strong evidence of discordance in genotype distribution between cases and controls due to possible miscalling were removed using an in-house Perl script.

To account for spurious associations resulting from ancestry differences among individuals, principal component (PC) analyses were performed in PLINK 1.9 and the gcta64 and R-base under GNU Public license V.2. We calculated the 10 first PCs using the markers informative of ancestry included in the Immunochip. Subjects showing more than four SDs from cluster centroids were excluded as outliers.

After applying quality control filters and genome imputation, we analyzed 252,970 polymorphisms in 37,159 autoimmune-disease patients (11,489 CeD, 15,523 RA, 3477 SSc, and 6670 T1D) and 22,308 healthy controls.

### Statistical analysis

#### Disease-specific analysis

First, we performed association analyses within each specific disease. For this, each case/control set was analyzed by logistic regression on the best-guess genotypes (> 0.9 probability) including the first ten PCs as covariates in PLINK 1.9. Then, for CeD, RA, and SSc, for which several independent case/control sets were available, we combined the different cohorts (Additional file 1: Table S1) using inverse variance weighted meta-analysis in METASOFT [13]. The human leukocyte antigen (HLA) region (Chr6: 20–40 MB) and sex chromosomes were excluded. Genomic inflation factor lambda (*λ*) was calculated using 3120 SNPs included in the Immunochip that map to non-immune regions. In addition, to account for inflation due to sample size [14], we calculated *λ*_1000_, the inflation factor for an equivalent study of 1000 cases and 1000 controls. Quantile–quantile plots for the *p* values of each individual disease are shown in Additional file 2: Figure S1a-d.

#### Cross-disease meta-analysis

Subsequently, summary level data obtained from the association studies of each specific disease were used to identify pleiotropic SNPs (shared by at least two of the autoimmune diseases analyzed). For this purpose, we performed a subset-based meta-analysis applying the “h traits” function as implemented in ASSET [15]. ASSET is an R statistical software package specifically designed for detecting association signals across multiple studies. This method does not only return a *p* value, but it also shows the best subset containing the studies contributing to the overall association signal. Moreover, this method allows for accounting for shared subjects across distinct studies using case/control overlap matrices. Since some of the control sets included in the disease-specific association analyses were shared among different diseases, we used correlation matrices to adjust for the overlapping of control individuals. Quantile–quantile plot for the *p* values from the cross-disease meta-analysis is shown in Additional file 2: Figure S1e.

After subset-based meta-analysis, SNPs for which two-tailed *p* values were lower than 5 × 10^− 8^ were considered statistically significant. Genetic variants showing effects in opposite directions across diseases were considered as significant when *p* values for both positively and negatively associated subsets reached at least nominal significance (*p* < 0.05). For regions where several SNPs reached genome-wide significance, we considered as lead variants those for which the best subset included a higher number of diseases. Subsequently, in order to identify independent signals, we linkage disequilibrium (LD)-clumped the results of the subset-based meta-analysis using PLINK to select polymorphisms with *r*^2^ < 0.05 within 500-kb windows and at genome-wide significant level.

#### Confirmation of pleiotropic effects identified by ASSET

To assess the reliability of our findings, ASSET results were compared with those obtained using an alternative approach, the compare and contrast meta-analysis (CCMA) [16]. For pleiotropic variants identified using ASSET, we calculated z-scores for each disease-specific association analysis as well as for all the possible combinations of diseases, assuming an agonistic or an antagonistic effect of the variants. For each locus, the subset showing the largest z-score was considered as the best model. *p* values for the maximum z-scores were derived using an empirical null distribution by simulating 300,000,000 realizations of four normally distributed random variables (*p* value < 1.00E−08 for z-score ≥ 6.45) (Additional file 2: Figure S2) [16].

#### Identification of novel genome-wide associations

We investigated whether pleiotropic SNPs were associated at genome-wide significance level with any of the diseases included in the best subset. To such purpose, we checked the results for these variants in each disease-specific association analysis. Additionally, in the case of SNPs associated with a specific disease, the statistical power of the subset-based analysis is lower than that of standard meta-analysis, as a result of a multiple-testing penalty associated with comprehensive subset searches. Consequently, the SNPs showing *p* values < 5 × 10^− 6^ in the subset-based meta-analysis were also tested for association in each specific disease.

#### Gene prioritization

To identify the most likely causal genes at associated *loci*, independent signals were annotated using several databases. First, all associated genetic variants were annotated using the variant effect predictor (VEP) [17]. Then, we used Immunobase [18] and the GWAS catalog [19] to explore whether the lead SNPs—or variants in LD with them (*r*^2^ ≥ 0.2) according to the European population of the 1000 Genomes Project—had been previously associated with immune-mediated diseases at genome-wide significance level. For SNPs for which clear candidate genes have already been reported, we considered these as the most probable genes. On the other hand, in the case of SNPs for which clear candidate genes have not been reported, we took into account VEP annotations, as follows: for SNPs annotated as coding, we reported the gene where each particular variant mapped; for SNPs annotated as intronic, upstream, downstream, or intergenic, we prioritized genes by using DEPICT (Data-driven Expression-Prioritized Integration for Complex Traits). DEPICT is an integrative tool that employs predicted gene functions to systematically prioritize the most likely causal genes at associated *loci* [20].

#### Functional annotation and enrichment analysis

Functional annotation of lead polymorphisms and their correlated variants (*r*^2^ ≥ 0.8) was performed using publicly available functional and biological databases. On the one hand, the possible functional impact of non-synonymous SNPs was evaluated using SIFT [21]. On the other hand, Haploreg v4.1 [22] was used to explore whether SNPs overlapped with conserved positions (Genomic Evolutionary Rate Profiling: GERP), tissue-specific chromatin state methylation marks (promoter and enhancer marks) based on the core-HMM 15 state model, tissue-specific DNase I hypersensitive sites (DHSs), tissue-specific transcription factor binding sites (TFBSs), and/or published expression quantitative trait *locus* (eQTL) signals in immune cell lines, cell types relevant for each specific disorder, and/or whole blood. Sources of Haploreg v4.1 include public datasets from the Roadmap Epigenomics project, the Encyclopedia of DNA Elements (ENCODE) Consortium and more than 10 eQTL studies, including the Genotype-Tissue Expression (GTEx) project.

Additionally, we used the GenomeRunner web server [23] to determine whether the set of pleiotropic SNPs significantly co-localized with regulatory genome annotation data in specific cell types from the ENCODE and Roadmap Epigenomics projects. Briefly, GenomeRunner calculates enrichment *p* values using Chi-squared test by evaluating whether a set of SNPs of interest co-localizes with regulatory datasets more often that could happen by chance. Specifically, we tested for overrepresentation of 161 TFBSs from the ENCODE project and histone modifications (acetylation of histone H3 at lysine 27 (H3K27ac), mono-methylation of histone H3 at lysine 4 (H3K4me1), and tri-methylation of histone H3 at lysine 4 (H3K4me3)) and DHSs in 127 cell types from the Roadmap Epigenomics project. Regulatory enrichment *p* values were corrected for multiple testing using the Benjamini–Hochberg false discovery rate (FDR) procedure.

#### Identification of common molecular mechanisms

Next, we performed protein-protein interaction (PPI) and pathway analysis to evaluate the existence of biological processes enriched among the set of pleiotropic *loci*. PPI analysis was conducted using STRING 10.5 [24], a database of direct (physical) and indirect (functional) interactions derived from five main sources: genomic context prediction, high-throughput lab experiments, co-expression, text mining, and previous knowledge in databases. In STRING, each PPI is annotated with a score, ranging from 0 to 1, which indicates the confidence of the interaction. We also used the list of common genes to perform KEGG pathway analysis using WebGestalt (WEB-based GEne SeT AnaLysis Toolkit) [25] with the human genome as reference set, the Benjamini Hochberg adjustment for multiple testing, and a minimum number of two genes per category.

#### Drug repurposing analysis

Finally, we investigated whether drugs currently used for other indications could be used for the treatment of RA, CeD, T1D, and/or SSc by using DrugBank (version 5.0.9, released 2017-10-02). DrugBank is a database containing 10,507 drug entries as well as 4772 non-redundant protein sequences linked to these drugs [26]. First, we identified genes in direct PPI with the pleiotropic genes by using STRING 10.5 [24], with a minimum required interaction score of 0.700 (high confidence) and excluding “text mining” as a source of interaction prediction. Subsequently, we searched DrugBank to identify pleiotropic genes, and genes in direct PPI with them, which are targets for approved, clinical trial or experimental pharmacologically active drugs.

## Results

### Cross-disease meta-analysis

After applying quality control filters and imputation, we analyzed Immunochip data from 37,159 patients diagnosed with an autoimmune disease (11,489 CeD, 15,523 RA, 3477 SSc, and 6670 T1D) and 22,308 healthy controls, all of them of European origin. We performed a subset-based association analysis using ASSET [15] to identify SNPs shared by at least two of the autoimmune conditions analyzed as well as the best subset of diseases contributing to the association signal. Summary statistics from the subset-based meta-analysis are available in Additional file 3. We observed 60 *loci* containing at least one genetic variant at genome-wide significance (*p* value ≤5 × 10^− 08^) in the meta-analysis (Additional file 2: Figure S3). After LD clumping, an independent association was found for 69 genetic variants within those genomic regions, 31 of which were associated with individual diseases and 38 were shared by two or more phenotypes (Additional file 1: Table S2).

The 38 identified common variants mapped on 34 different genomic regions (Table 1 and Additional file 1: Table S2). According to the GWAS Catalog and Immunobase [18, 19], five of these shared *loci* (*PADI4* at 1p36.13, *NAB1* at 2q32.3, *COBL* at 7p12.1, *CCL21* at 9p13.3, and *GATA3* at 10p14) have been associated with a single autoimmune disease so far and thus they represent new pleiotropic *loci* in autoimmunity. We also observed several independent signals within three known shared risk *loci*, four of which (rs1217403 in *PTPN22*, rs6749371 and rs7574865 in *STAT4*, and rs17753641 in *IL12A*) are new signals for some of the diseases contributing to the association (Table 1 and Additional file 1: Table S2). For example, we identified two independent variants associated with RA and T1D in *PTPN22*: rs2476601—a known risk variant for both conditions—and rs1217403—which is not linked to the SNPs previously associated with RA and T1D (*r*^2^ = 0.03). Interestingly, three independent multi-disease signals were detected within the 2q32.3 region, two of them (rs6749371 and rs7574865) located within *STAT4* and another one (rs10931468) located within the *NAB1* gene (Table 1 and Additional file 1: Table S2). Interestingly, this last *locus* has not been previously associated with any of the diseases contributing to the association signal, RA, and SSc.

**Table 1:** Independent genetic variants reaching genome-wide level of significance in the subset-based meta-analysis and showing pleiotropic effects across diseases

Region	Position (bp)	SNP	Gene	A1	P2sided	Best subset
1p36.32	2,534,978	rs6664969	*MMEL1*	A	2.86E−10	CeD RA
1p36.13	17,655,407	rs1748041	*PADI4*	C	3.63E−08	RA **SSc**
1p13.2	114,377,568	rs2476601	*PTPN22*	A	6.36E−119	RA T1D
1p13.2	114,388,804	rs1217403	*PTPN22*	C	4.66E−11	RA* T1D*
1q24.3	172,674,776	rs10912267	*FASLG*	A	3.90E−09	CeD **T1D**
2q11.2	100,764,004	rs13415465	*AFF3*	G	3.72E−12	**CeD** RA T1D
2q31.3	182,057,640	rs12619531	*ITGA4*	G	1.18E−18	CeD **SSc**
2q32.3	191,538,562	rs10931468	*NAB1*	A	1.56E−08	**RA SSc**
2q32.3	191,902,184	rs6749371	*STAT4*	T	3.84E−08	CeD SSc*
2q32.3	191,964,633	rs7574865	*STAT4*	T	3.16E−09	CeD* RA SSc T1D*
2q33.2	204,612,058	rs7426056	*CD28*	A	6.68E−12	CeD RA
2q33.2	204,738,919	rs3087243	*CTLA4*	A	5.08E−16	RA T1D
3p14.3	58,183,636	rs35677470	*DNASE1L3*	A	1.04E−11	RA SSc
3q25.33	159,647,674	rs17753641	*IL12A*	G	1.64E−29	CeD SSc*
4p15.2	26,088,128	rs16878091	*RBPJ*	A	2.53E−12	RA T1D
5q33.1	150,438,988	rs1422673	*TNIP1*	T	1.87E−09	**CeD RA** SSc
6q15	90,976,768	rs72928038	*BACH2*	A	9.34E−12	CeD RA T1D
6q23.3	138,003,822	rs11757201	*TNFAIP3*	C	1.27E−11	CeD RA T1D
6q23.3	138,243,739	rs58721818	*TNFAIP3*	T	5.26E−10	RA SSc
6q25.3	159,470,417	rs212407	*TAGAP*	G	6.74E−14	CeD RA T1D
7p14.1	37,382,465	rs60600003	*ELMO1*	G	4.25E−13	CeD **SSc**
7p12.1	51,015,193	rs7780389	*COBL*	T	2.25E−08	**RA** T1D
7q32.1	128,572,766	rs4731532	*IRF5*	A	1.25E−10	RA SSc
9p13.3	34,710,260	rs2812378	*CCL21*	G	1.04E−09	**CeD** RA
10p15.1	6,101,713	rs3118470	*IL2RA*	C	5.92E−09	RA T1D
10p15.1	6,116,254	rs72776098	*IL2RA*	A	7.10E−10	**SSc** T1D
10p15.1	6,390,450	rs947474	*PRKCQ*	G	1.28E−08	CeD RA T1D
10p14	8,102,272	rs3802604	*GATA3*	G	4.67E−08	RA **T1D**
10q22.3	81,045,280	rs1250568	*ZMIZ1*	C	3.87E−15	CeD **SSc T1D**
11q23.3	118,726,843	rs10892299	*DDX6*	T	2.25E−13	CeD **SSc T1D**
12q13.2	56,470,625	rs11171739	*IKZF4*	C	1.87E−20	RA T1D
15q14	38,828,140	rs8043085	*RASGRP1*	T	1.53E−08	RA T1D
15q25.1	79,234,957	rs34593439	*CTSH*	A	1.47E−14	CeD T1D
17q12	38,033,277	rs1054609	*ORMDL3*	C	3.70E−08	RA SSc T1D
18p11.21	12,777,573	rs2542148	*PTPN2*	C	5.11E−16	CeD T1D
19p13.2	10,427,721	rs74956615	*TYK2*	A	1.62E−17	RA SSc T1D
21q22.3	43,855,067	rs1893592	*UBASH3A*	C	4.86E−12	CeD T1D
22q11.1	21,936,152	rs66534072	*YDJC*	G	2.05E−08	CeD **SSc**

The selected lead SNP in each region is shown, together with the best subset obtained from the subset-based meta-analysis. Position (bp), base pair position in hg19; SNP, single nucleotide polymorphism; Gene, annotated gene as described in methods; A1, alternative allele used in the logistic regression; P2sided, *p* value from the two-sided subset-based meta-analysis; Best subset, phenotypes contributing to the association signal. Diseases included in the best subset and for which identified associations have not been previously reported are shown in bold; novel signals within known risk *loci* are indicated by “*”

On the other hand, an opposite effect was observed for ten of the shared genetic variants that mapped on *ITGA4*, *IL12A*, *TNIP1*, *TAGAP*, *COBL*, *IL2RA*, *ZMIZ1*, *DDX6*, *IKZF4*, and *CTSH* regions (Additional file 2: Figure S4 and Table S3). For example, the minor allele (G) of the *IL12A* rs17753641 polymorphism, which has been previously reported to confer risk to CeD, had a protective effect for SSc in our study. In addition, an opposite effect was also observed for the *TAGAP* rs212407 variant, which appeared to confer risk to CeD and protection to RA and T1D, as previously described [6, 27].

In order to validate our findings, the pleiotropic role of the shared variants identified by ASSET was evaluated using the CCMA approach. As shown in Additional file 1: Table S4, 34 of the 38 SNPs had a pleiotropic effect according to CCMA (best model including at least two diseases). It should be noted that the second best model obtained with this method yielded z-scores very similar to those of the best model. In this regard, when considering either of the two best models, all pleiotropic SNPs identified by ASSET showed shared effects across diseases in the CCMA (Additional file 1: Table S4). Furthermore, we observed a high concordance rate between the best subset of diseases identified by ASSET and the best models (best or second best model) according to CCMA. Specifically, best models completely matched between both methods for 29 of the 38 SNPs (concordance rate of 0.76). In addition, for the remaining 9 pleiotropic variants, best models partially overlapped between ASSET and CCMA and, in all the cases except one, diseases contributing to the association signal according to ASSET were included in the best model of CCMA (Additional file 1: Table S4). For instance, whereas ASSET identified two diseases (CeD and SSc) contributing to the association signal observed for rs60600003, the best model obtained with CCMA included three diseases, the two already forming part of the best subset of ASSET (CeD, SSc) and RA. Considering those SNPs for which the best model overlapped totally or partially between both approaches, the concordance rate between ASSET and CCMA was 0.87, considering the best model of CCMA, and 1, considering the best or second best model of CCMA. This analysis confirms the high reliability of our cross-disease meta-analysis results, strongly supporting the role of the 38 genetic variants as pleiotropic risk factors in autoimmunity.

### Identification of novel individual-disease associations

**Fig. 1:**
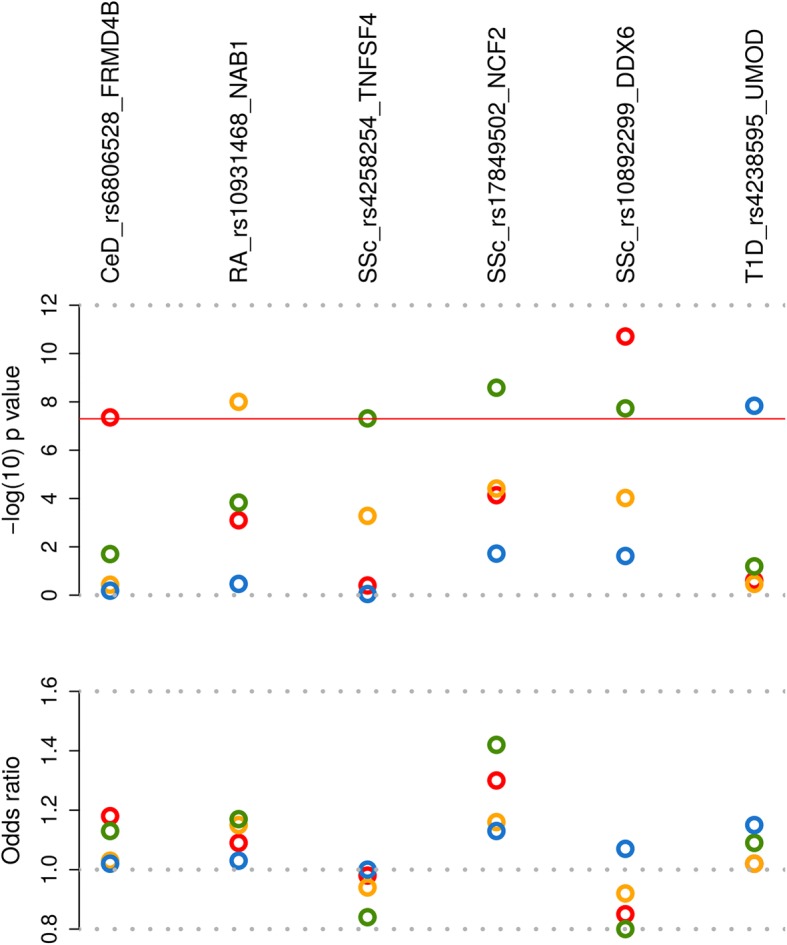
Novel genome-wide associated *loci* for celiac disease, rheumatoid arthritis, systemic sclerosis and type 1 diabetes. Pleiotropic SNPs reaching genome-wide significance level and SNPs associated with a single disease and reaching *p* values lower than 5 × 10^− 6^ in the subset-based meta-analysis were checked for genome-wide association in each of the diseases included in the best subset. Negative log_10_-tranformed *p* value (disease-specific *p* values) (upper plot) and odds ratio (lower plot) for the new genome-wide signals are shown. The six *loci* are annotated with the candidate gene symbol. Circles represent the analyzed diseases (red: celiac disease; yellow: rheumatoid arthritis; green: systemic sclerosis; blue: type 1 diabetes). The red line represents genome-wide level of significance (*p* = 5 × 10^− 8^)

Of the 34 shared risk *loci* identified, 20 have already been reported as risk factors for the diseases contributing to the association, according to Immunobase and the GWAS catalog [18, 19], whereas 14 of them (more than 40%) represent potentially new *loci* for at least one of the diseases included in the best subset (Table 1). Considering this, we checked whether these pleotropic variants were associated at genome-wide level of significance with any of the diseases contributing to each specific signal. Two of the common variants, rs10931468 (mapping on the *NAB1* region, 2q32.3) and rs10892299 (mapping on the *DDX6* region, 11q23.3), were associated with RA and SSc, respectively (Fig. 1, Additional file 2: Figures S5a and S6a, and Additional file 1: Table S2); hence they represent novel genetic risk factors for these diseases. The rs10931468 genetic variant is located within the *NAB1* gene, near *STAT4* (Table 1). However, this SNP is not linked to the *STAT4* variants previously associated with the diseases under study (*D*’ < 0.13 and *r*^2^ < 0.012). In fact, this SNP showed an independent effect in the RA meta-analysis after conditioning on the most associated variants within the region (Additional file 2: Figure S5b).

In addition, to avoid any loss of power, SNPs associated with a single disease and reaching *p* values lower than 5 × 10^− 6^ in the subset-based meta-analysis were checked for association in each specific disorder. Using this strategy, we identified four novel single-disease genome-wide associations, one for CeD (rs6806528 at *FRMD4B*), two for SSc (rs4258254 at *TNFSF4* and rs17849502 at *NCF2*), and one for T1D (rs4238595 at *UMOD*) (Fig. 1, Additional file 2: Figures S6-S8, and Additional file 1: Table S5).

### Functional annotation of associated variants

SNP annotation showed that only 5% of the pleiotropic SNPs were coding, including two missense variants (Additional file 1: Table S2), whereas five of the non-coding SNPs (13%) were in tight LD (*r*^2^ ≥ 0.8) with coding variants (three missense, one synonymous and one splice donor) (Additional file 2: Table S6). Two of the non-synonymous polymorphisms, rs35677470 within *DNASE1L3* and rs2289702 (a proxy for rs34593439) within *CTSH*, appeared to have a deleterious effect according to SIFT (Additional file 1: Table S2). Of the four new single-disease signals, three were non-coding polymorphisms and one was a missense variant (Additional file 1: Table S5).

**Fig. 2:**
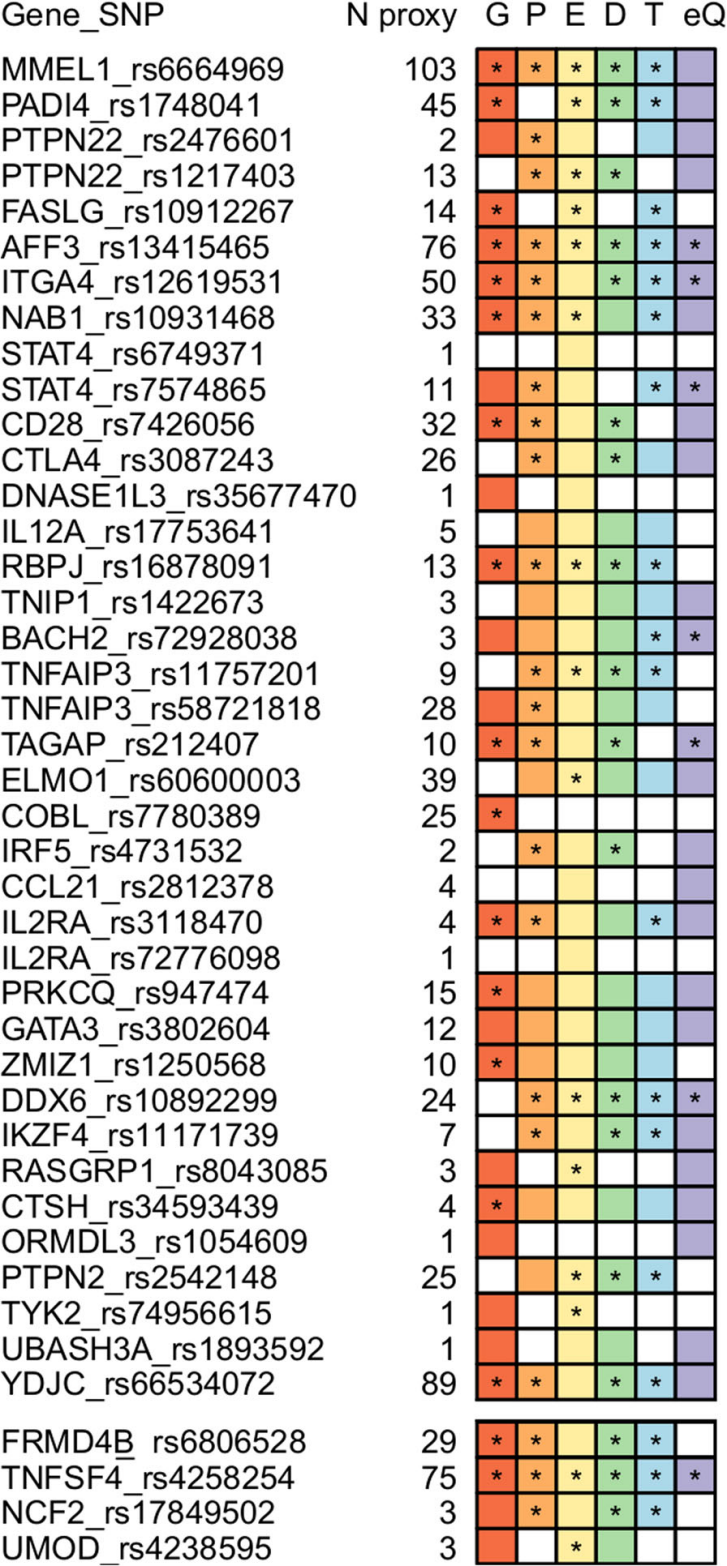
Functional annotation of 38 pleiotropic polymorphisms (*p* < 5 × 10^–8^ in the subset-based meta-analysis) and four single-disease associated variants (*p* < 5 × 10^–6^ in the subset-based meta-analysis and *p* < 5 × 10^–8^ in disease-specific meta-analyses). Haploreg v4.1 was used to explore whether lead SNPs, and their proxies (*r*^2^ ≥ 0.8), overlapped with different regulatory datasets from the Roadmap Epigenomics project, the ENCODE Consortium and more than ten eQTL studies in immune cell lines, cell types relevant for each specific disorder and/or whole blood. Colors denote both lead and proxy SNPs overlapping with the different regulatory elements analyzed: G (red): conserved positions (Genomic Evolutionary Rate Profiling, GERP); P (orange): promoter histone marks; E (yellow): enhancer histone marks; D (green): DNase I hypersensitive sites (DHS); T (blue): transcription factor binding sites (TFBSs); eQ (purple): expression quantitative trait *loci* (eQTL). Functional annotations overlapping with proxy SNPs are marked with an asterisk. N proxy, number of proxy SNPs for each lead variant. The different *loci* are annotated with the candidate gene symbol

Considering that most of the associated genetic variants did not show direct effects on protein function, we identified all SNPs in high LD (*r*^2^ ≥ 0.8) with both pleiotropic and single-disease lead signals and evaluated their possible functional implications. We checked for overlap between the lead and proxy SNPs and functional annotations from the Roadmap Epigenomics, ENCODE and GTEx projects, including conserved positions, histone modifications at promoters and enhancers, DHS, TFBS, and eQTL. As shown in Fig. 2, all pleiotropic SNPs lie in predicted regulatory regions in immune cell lines or whole blood, whereas 76% overlap with more than three functional annotations. In addition, most of them appear to act as eQTLs, thereby affecting gene expression levels (Fig. 2 and Additional file 1: Table S7).

Similarly, all single-disease-associated variants also overlapped with regulatory elements in whole blood, immune cells, and/or cell types relevant for each specific disorder (Fig. 2 and Additional file 1: Table S7).

### Enrichment in tissue-specific regulatory elements and biological pathways

**Fig. 3:**
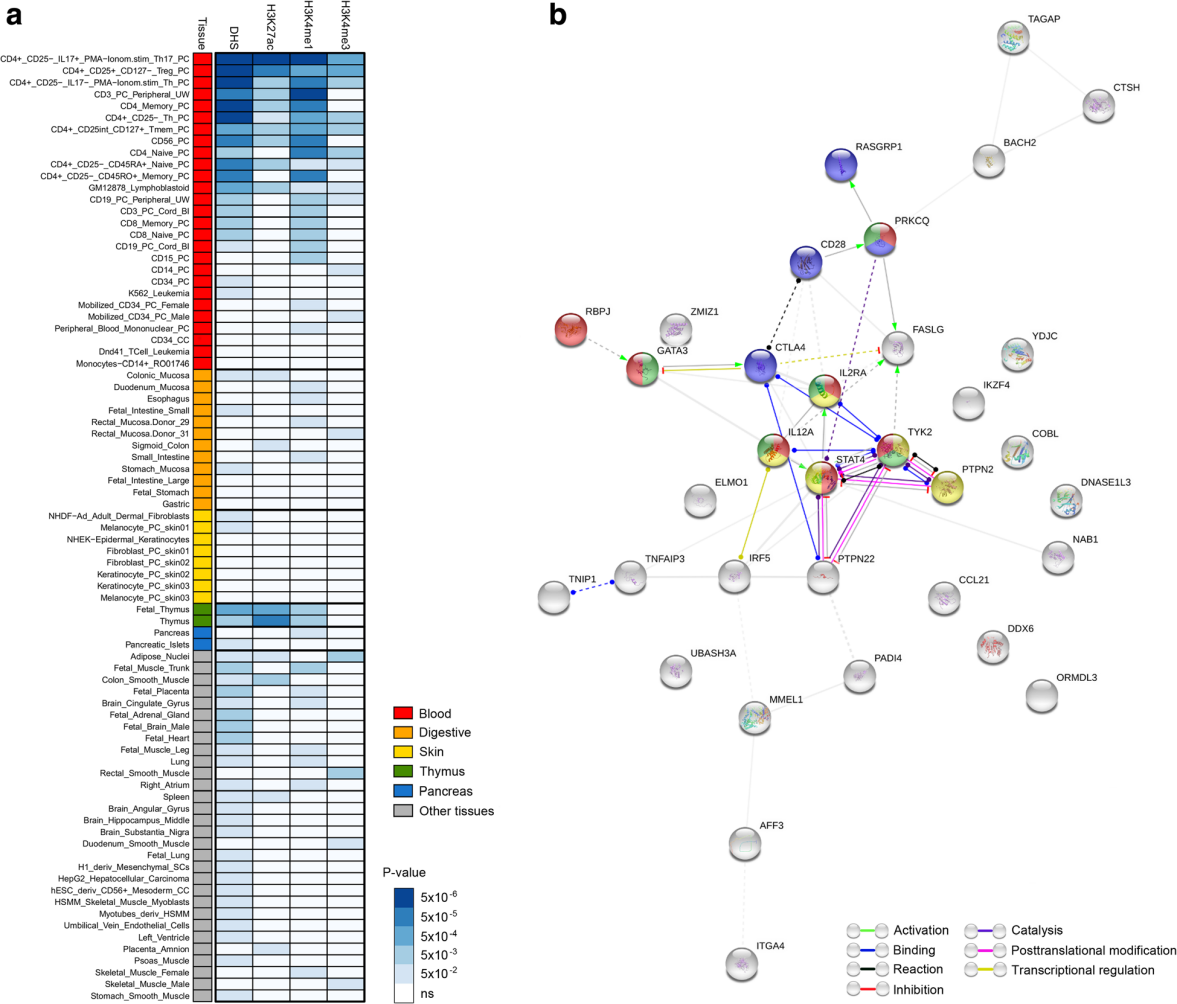
Functional regulatory elements and PPI enrichment analysis. **a** Heat map showing DNase 1 hypersensitive sites (DHSs) and histone marks enrichment analysis of the set of pleiotropic variants. GenomeRunner web server was used to determine whether the set of pleiotropic SNPs significantly co-localize with regulatory genome annotation data in 127 cell types from the Roadmap Epigenomics project. First column shows cell types grouped and colored by tissue type (color-coded as indicated in the legend). Tissues relevant for the autoimmune diseases studied as well as other tissues for which any of the analyzed functional annotations showed a significant enrichment *p* value (*p* < 0.05 after FDR correction) are shown. The remaining four columns denote the analyzed functional annotations, DHSs, H3K27ac, H3K4me1, and H3K4me3. Results of the enrichment analysis are represented in a scale-based color gradient depending on the *p* value. Blue indicates enrichment and white indicates no statistical significance after FDR adjustment. **b** Interaction network formed for the set of common genes. Direct and indirect interactions among genes shared by different disease subgroups were assessed using STRING. Plot shows results of the “molecular action” view such that each line shape indicates the predicted mode of action (see legend). Genes involved in the biological pathways enriched among the set of pleiotropic *loci* (Additional file 2: Table S10) are shown in color: red: Th1 and Th2 cell differentiation; green: Th17 cell differentiation; yellow: Jak-STAT signaling pathway; blue: T cell receptor signaling pathway

Subsequently, to determine whether the set of 38 independent pleiotropic SNPs was enriched for regulatory elements in specific cell types, we performed a hypergeometric test using GenomeRunner [23]. Specifically, we checked for overrepresentation of DHSs, histone modifications (H3K27ac, H3K4me1, and H3K4me3), and TFBSs in human cell lines and tissues from the ENCODE and Roadmap Epigenomics projects. Results of this analysis are shown in Fig. 3a and Additional file 1: Table S8. Pleiotropic SNPs showed overrepresentation of DHSs in different subsets of T cells, with the strongest enrichment pointing to regulatory T (Treg) cells, T helper memory and naive cells, and Th17 lymphocytes. Similarly, the H3k4me1, H3k27ac, and H3k4me3 histone marks—which are especially informative of most active enhancer and promoter regulatory regions—were also overrepresented in these specific cell types (Fig. 3a and Additional file 1: Table S8). In addition, shared genetic variants were enriched for targets of 12 TFs, with BATF (P_BH_ = 6.40E−15), RelA (*P*_BH_ = 6.11E−12), and IRF4 (*P*_BH_ = 1.88E−08) showing the strongest overrepresentation (Additional file 2: Table S9).

We further conducted PPI and KEGG pathway analysis to gain insight into the biological processes affected for the set of common genes. By constructing a network of direct and indirect interactions, we found a main cluster enriched for proteins involved in Th1 and Th2 cell differentiation (*P*_BH_ = 6.21E−07), Jak-STAT signaling pathway (*P*_BH_ = 4.53E−03), T cell receptor signaling pathway (*P*_BH_ = 7.85E−03), and Th17 cell differentiation (*P*_BH_ = 7.85E−03) (Fig. 3b and Additional file 2: Table S10).

### Identification of potential drug targets

Finally, in order to identify potentially new leads for therapies for CeD, RA, SSc, and T1D, we investigated whether proteins encoded by pleiotropic genes—or any gene in direct PPI with them—are targets for approved, clinical trial, or experimental pharmacologically active drugs. Using this approach, we found 26 potentially repositionable drugs: 8 indicated for RA that would be worth exploring for CeD, SSc, and/or T1D treatment and 18 with other indications that could be promising candidates for the treatment of at least two of the four autoimmune diseases under study (Table 2). Interestingly, 15 of the 19 drug targets identified among the set of common genes are involved in the biological pathways overrepresented in the set of autoimmune disease common genes (Fig. 3b).

**Table 2:** Common genes in autoimmunity identified as targets for drugs

Annotated gene	Genes in direct PPI	Targeted drugs	Action	Indication	Potential new clinical application
Indicated for CeD, RA, T1D, and/or SSc					
*CD28*	*CD80*	Abatacept	Antagonist	RA	CeD
*IL12A/TYK2*	*IL6R*	Tocilizumab	Antibody	RA	CeD, SSc, T1D
		Sarilumab	Antagonist, antibody	RA	
	*IL1R1*	Anakinra	Antagonist	RA	
*PTPN2/STAT4*	*JAK1/JAK2/JAK3*	Tofacitinib	Inhibitor	RA	CeD, SSc, T1D
*TNFAIP3*	*TNF*	Etanercept	Antibody	RA	CeD, SSc, T1D
		Adalimumab	Antibody	RA	
		Infliximab	Inhibitor	RA	
Other indications					
*CD28*	*CD2*	Alefacept	Inhibitor	Psoriasis	CeD, RA
*CD28/IL12A/IL2RA/STAT4/TYK2*	*IFNG*	Olsalazine	NA	Inflammatory bowel disease	CeD, RA, SSc, T1D
*CCL21*	*C5*	Eculizumab	Antibody	Paroxysmal nocturnal haemoglobinuria	CeD, RA
	*CXCR4*	Plerixafor	Antagonist	Cancer	
*CCL21/IL12A/TYK2*	*CCR5*	Maraviroc	Antagonist	HIV	CeD, RA, SSc, T1D
*CTLA4*		Ipilimumab	NA	Cancer	RA, T1D
*FASLG/IL12A/IL2RA/IRF5/STAT4/TYK2*	*IL12B*	Ustekinumab	Antibody	Psoriasis and psoriatic arthritis	CeD, RA, SSc, T1D
*IL12A/IL2RA/TYK2*	*IL3RA*	Sargramostim	Agonist	Cancer	CeD, RA, SSc, T1D
*IL12A/IRF5/TYK2*	*IL1B*	Canakinumab	Binder	Systemic juvenile idiopathic arthritis	CeD, RA, SSc, T1D
*IL12A/TYK2*	*IFNGR1*	Interferon gamma-1b		Chronic granulomatous disease	CeD, RA, SSc, T1D
*IL2RA*		Aldesleukin	Agonist, Modulator	Cancer	CeD, RA, SSc, T1D
		Basiliximab	Antibody	Kidney transplant rejection	
		Daclizumab	Antibody	Multiple sclerosis	
		Denileukin diftitox	Binder	Cancer	
*IL2RA/IRF5/TYK2*	*IL6*	Siltuximab	Antagonist antibody	Castleman’s disease	CeD, RA, SSc, T1D
*IL2RA/STAT4/TYK2*	*IL23A*	Guselkumab	Blocker	Psoriasis	CeD, RA, SSc, T1D
*ITGA4*		Natalizumab	Antibody	Multiple sclerosis	CeD, SSc
		Vedolizumab	Antibody	Crohn disease and ulcerative colitis	

Target genes for both drugs used for the treatment of the studied autoimmune diseases as well as drugs used for other indications are shown in the Table. NA, not available. Last column indicates those diseases that could potentially benefit from drug repositioning, since they are contributing (included in the best subset) to the association signal/s observed within each *locus*

## Discussion

Through a large cross-disease meta-analysis of Immunochip data from four seropositive autoimmune disorders, CeD, RA, SSc, and T1D, we have been able to advance in the knowledge of the genetic overlap existing in autoimmunity. Specifically, our meta-analysis identified 38 genetic variants shared among subsets of the diseases under study, five of which, including *PADI4*, *NAB1*, *COBL*, *CCL21*, and *GATA3*, represent new shared genetic risk *loci*. Moreover, ten of the 38 pleiotropic variants showed opposite allelic effects across phenotypes contributing to the association signal, thus indicating the complexity of the molecular mechanisms by which SNPs affect autoimmune diseases.

Consistent with previous findings [28], functional annotation of these pleiotropic polymorphisms suggested that the majority of multi-disease signals affect disease risk by altering gene regulation. Interestingly, tissue-specific enrichment analysis for regulatory elements suggested a specific regulatory role of the pleiotropic variants in Th17 and Treg cells, thus pointing to a crucial contribution of these cell types to the pathogenic mechanisms shared by these disorders. In addition, enrichment for targets of several TFs, mainly BATF, RelA, and IRF4, was also evident. It should be noted that BATF and IRF4 are both required for the differentiation of Th17 cells [29], whereas RelA is crucial for Treg-induced tolerance [30]. According to this data, pleiotropic variants could potentially regulate gene expression by disrupting motifs recognized for TFs in different subsets of T cells, mainly Th17 and Treg lymphocytes. Subsequently, results from pathway enrichment analysis confirmed the relevant contribution of pleiotropic variants and target genes in T cell-mediated immunity. Moreover, drug repositioning analysis evidenced several candidate drugs with potential new clinical use for the diseases under study. Notably, most of these drugs were directed against proteins involved in the biological processes overrepresented among the set of common genes and, therefore, their potential clinical application to the treatment of CeD, RA, SSc, and T1D appeared to be of special interest. However, it should be considered that both the functional effects of pleiotropic variants as well as the disease-causal genes remain elusive in most cases, thus representing a limitation for drug repositioning. In addition, ten of these shared genetic variants showed opposite effects across diseases and, therefore, the complexity of molecular mechanisms by which SNPs affect autoimmune diseases should be taken into account when prioritizing drugs based on repositioning studies.

Furthermore, we also reported six new genome-wide associations for the diseases under study. We identified two new susceptibility *loci* for RA and SSc among the pleiotropic signals. The dense genotyping of immune-related *loci* provided by the Immunochip platform allowed identifying *NAB1* as a new susceptibility *locus* for RA within the 2q22.3 region, which also contains the pan-autoimmune susceptibility gene *STAT4*. In addition, interrogation of publicly available eQTL data sets showed that the associated *NAB1* variant, rs10931468, acts as an eQTL affecting *NAB1* expression in lymphoblastoid cell lines. *NAB1* encodes the NGFI-A binding protein 1, which has been shown to form a complex with Egr3 involved in the silencing of interferon gamma receptor 1 (ifngr1). Specifically, Nab1 was required for deacetylation of the ifngr1 promoter and downregulation of cell surface receptor [31]. On the other hand, an intergenic variant located near *DDX6* was also identified as a new genetic risk *locus* for SSc. This gene encodes a member of the DEAD box protein family recently identified as a suppressor of interferon-stimulated genes [32].

Additionally, some of the single-disease genome-wide associations identified in the present study had not been previously reported. The *FRMD4B* locus was found to be associated with CeD. Although genetic variants within the *FRMD4B* region have been previously involved in disease susceptibility [33, 34], our study is the first one reporting an association between CeD and this *locus* at the genome-wide significance level. *FRMD4B*, encoding a scaffolding protein (FERM domain containing 4B protein), has not been described before in relation to any autoimmune disorder, representing a CeD-specific risk *locus*.

Regarding SSc, two new genetic risk *loci* were identified. According to the subset-based meta-analysis results, SSc was the only phenotype contributing to the association signal detected within the 1q25.1 region; however, this *locus* is also a known susceptibility factor for RA [35]. Indeed, several SNPs within this region showed pleiotropic effects in RA and SSc in the cross-disease meta-analysis, but they did not reach genome-wide significance (top RA-SSc common signal: *p* value = 5.86E−06). A relevant gene for the immune response, *TNFSF4*, is located within the 1q25.1 region; nevertheless, functional annotation revealed that the rs10798269 SNP (a proxy for the top associated variant) acted as a trans-eQTL influencing the expression level of the *PAG1* gene (*p* value = 4.20E−06). Strikingly, *PAG1*, residing on chromosome region 8q21.13, encodes a transmembrane adaptor protein that binds to the tyrosine kinase csk participating in the negative control of the signaling mediated by the T cell receptor (TCR) [36]. It should be noted that *CSK* is an established risk *locus* for SSc [37]. A second novel genome-wide association for SSc was identified within the 1q25.3 region. The strongest signal belonged to a missense variant (rs17849502), also associated with systemic lupus erythematosus [38], which leads to the substitution of histidine-389 with glutamine (H389Q) in the PB1 domain of the neutrophil cytosolic factor 2 (NCF2) protein. NCF2 is part of the multi-protein NADPH oxidase complex found in neutrophils. Interestingly, it has been shown that the 389Q mutation has a functional implication, causing a twofold decrease in reactive oxygen species production [38].

Finally, a genetic variant (rs4238595) located downstream of the *UMOD* gene, encoding uromodulin, was identified as a new genetic risk factor for T1D. Interestingly, a SNP linked to this variant showed nominal association in a previous GWAS performed in this disorder [39]. This *locus* has also been implicated in diabetic kidney disease [40]. Nevertheless, no association with any other immune-related condition has been described so far and, therefore, this *locus* represents a T1D-specific association. In addition, functional annotation of the lead variant and their proxies showed an overlap with enhancer histone marks and DHSs specifically in pancreas, which supports its potential role in the T1D pathogenesis.

## Conclusions

In summary, by conducting a subset-based meta-analysis of Immunochip data from four seropositive autoimmune diseases, we have increased the number of pleiotropic risk *loci* in autoimmunity, identified new genome-wide associations for CeD, SSc, RA, and T1D and shed light on common biological pathways and potential functional implications of shared variants. Knowledge of key shared molecular pathways in autoimmune diseases may help identify putative common therapeutic mechanisms. In this regard, we identified several drugs used for other indications that could be repurposed for the treatment of the autoimmune diseases under study. Thus, a new classification of patients based on molecular profiles, rather than clinical manifestations, will make it possible for individuals with a certain autoimmune disorder to benefit from therapeutic options currently used to treat another disease with which they share etiological similarities.

Due to the design of the Immunochip, all shared pathways identified in our study were related to immune regulation. Hopefully, future cross-disease studies using GWAS data will allow identification of non-immune *loci* and pathways shared in autoimmunity.

## Additional files

Additional file 1: **Table S1.** Case/control datasets included in the study. **Table S2. ***Loci* reaching genome-wide level of significance in the subset-based meta-analysis and showing independent effect after linkage disequilibrium (LD)-clumping (*r*^2^ < 0.05 within 500 kB up- or downstream of the lead SNP). **Table S4.** Comparison of the results obtained with ASSET and CCMA for the 38 pleiotropic variants identified in our study. **Table S5.** Novel genome-wide associations for celiac disease, systemic sclerosis and type 1 diabetes (*p* value < 5 × 10–6 in the subset based meta-analysis and *p* value < 5 × 10–8 in each disease-specific meta-analysis). **Table S7.** Potential role of the lead polymorphisms (pleiotropic and single-disease associated variants), and their proxies (*r*^2^ ≥ 0.8) as expression quantitative trait loci (eQTLs) in whole blood, immune cell lines or tissues relevant for the diseases under study. **Table S8.** Specific cell types showing enrichment among regulatory DNA elements, Dnase 1 hypersensitivity sites and histone marks, and pleiotropic variants. (XLSX 77 kb)

Additional file 2: **Table S3.** Results of the subset-based meta-analysis for the lead variants showing evidence of opposite allelic effect across the autoimmune diseases contributing to the association signal. **Table S6.** Coding variants in tight linkage disequilibrium (*r*^2^ ≥ 0.8) with lead non-coding polymorphisms according to the European population of the 1000 Genomes Project. **Table S9.** Transcription factor binding sites (TFBSs) potentially disrupted by the set of pleiotropic variants. **Table S10.** Biological pathways significantly enriched among the set of common genes. **Figure S1.** Quantile–quantile plots for the *p* values of each individual disease, celiac disease (a), rheumatoid arthritis (b), systemic sclerosis (c), and type 1 diabetes (d), and the cross disease meta-analysis (e). **Figure S2.** Empirical −log10(P)-distribution of the Zmax statistic obtained by simulating 300 × 10^6^ replicates of four normally distributed random variables. **Figure S3.** Manhattan plot of the subset-based meta-analysis of Immunochip data from celiac disease (CeD), systemic sclerosis (SSc), rheumatoid arthritis (RA) and type 1 diabetes (T1D). **Figure S4.** Disease-specific odds ratio for the pleiotropic variants showing opposite allelic effects across autoimmune diseases. **Figure S5.** Regional association plots of the novel genome-wide associated locus for rheumatoid arthritis (RA), 2q32.3. **Figure S6.** Regional association plots of the novel genome-wide associated *loci* for systemic sclerosis (SSc), 11q23.3 (a), 1q25.1 (b), and 1q25.3 (c). **Figure S7.** Regional association plot of the novel genome-wide associated locus for celiac disease (CeD), 3p14.1. **Figure S8.** Regional association plot of the novel genome-wide associated locus for type 1 diabetes (T1D), 16p12.3. Members of the Coeliac Disease Immunochip Consortium, Members of the RACI, Members of the International Scleroderma Group, Members of the Type 1 Diabetes Genetics Consortium (T1DGC). (PDF 1590 kb)

Additional file 3: Summary statistics from the cross-disease meta-analysis using ASSET. (TXT 38863 kb)

## Abbreviations

ACSL4: Acyl-CoA synthetase long chain family member 4
BATF: Basic leucine zipper ATF-like transcription factor
CCL21: C–C motif chemokine ligand 21
CeD: Celiac disease
COBL: Cordon-bleu WH2 repeat protein
CSK: C-terminal Src kinase
CTSH: Cathepsin H
DDX6: DEAD-box helicase 6
DHS: DNase I hypersensitive site
DNASE1L3: Deoxyribonuclease 1 like 3
eQTL: Expression quantitative trait locus
FDR: False discovery rate
FRMD4B: FERM domain containing 4B
GATA3: GATA binding protein 3
GERP: Genomic Evolutionary Rate Profiling
GWAS: Genome-wide association study
H3K27ac: Acetylation of histone H3 at lysine 27
H3K4me1: Mono-methylation of histone H3 at lysine 4
H3K4me3: Tri-methylation of histone H3 at lysine 4
HLA: Human leukocyte antigen
IL12A: Interleukin 12A
IRF4: Interferon regulatory factor 4
Jak: Janus kinase
KEEG: Kyoto Encyclopedia of Genes and Genomes
LD: Linkage disequilibrium
NAB1: NGFI-A binding protein 1
NCF2: Neutrophil cytosolic factor 2
PADI4: Peptidyl arginine deiminase 4
PAG1: Phosphoprotein membrane anchor with glycosphingolipid microdomains 1
PC: Principal component
PPI: Protein-protein interaction
PTPN22: Protein tyrosine phosphatase, non-receptor type 22
RA: Rheumatoid arthritis
RelA: RELA proto-oncogene, NF-kB subunit
SD: Standard deviation
SLC22A5: Solute carrier family 22 member 5
SNP: Single-nucleotide polymorphism
SSc: Systemic sclerosis
STAT4: Signal transducer and activator of transcription 4
T1D: Type 1 diabetes
TAGAP: T cell activation RhoGTPase activating protein
TF: Transcription factor
TFBS: Transcription factor binding site
TNFSF4: TNF superfamily member 4
Treg: Regulatory T cell
UMOD: Uromodulin
