# Supplementary material for: Meta-analysis of Immunochip data of four autoimmune diseases reveals novel single-disease and cross-phenotype associations
Source: Genome Med. 2018 Dec 20;10:97. doi: 10.1186/s13073-018-0604-8 (PMC6302306; doi:10.1186/s13073-018-0604-8)
Supplement: Supplementary file 2 — Table S3. Results of the subset-based meta-analysis for the lead variants showing evidence of opposite allelic effect across the autoimmune diseases contributing to the association signal. Table S6. Coding variants in tight linkage disequilibrium (r2 ≥ 0.8) with lead non-coding polymorphisms according to the European population of the 1000 Genomes Project. Table S9. Transcription factor binding sites (TFBSs) potentially disrupted by the set of pleiotropic variants. Table S10. Biological pathways significantly enriched among the set of common genes. Figure S1. Quantile–quantile plots for the p values of each individual disease, celiac disease (a), rheumatoid arthritis (b), systemic sclerosis (c), and type 1 diabetes (d), and the cross disease meta-analysis (e). Figure S2. Empirical −log10(P)-distribution of the Zmax statistic obtained by simulating 300 × 106 replicates of four normally distributed random variables. Figure S3. Manhattan plot of the subset-based meta-analysis of Immunochip data from celiac disease (CeD), systemic sclerosis (SSc), rheumatoid arthritis (RA) and type 1 diabetes (T1D). Figure S4. Disease-specific odds ratio for the pleiotropic variants showing opposite allelic effects across autoimmune diseases. Figure S5. Regional association plots of the novel genome-wide associated locus for rheumatoid arthritis (RA), 2q32.3. Figure S6. Regional association plots of the novel genome-wide associated loci for systemic sclerosis (SSc), 11q23.3 (a), 1q25.1 (b), and 1q25.3 (c). Figure S7. Regional association plot of the novel genome-wide associated locus for celiac disease (CeD), 3p14.1. Figure S8. Regional association plot of the novel genome-wide associated locus for type 1 diabetes (T1D), 16p12.3. Members of the Coeliac Disease Immunochip Consortium, Members of the RACI, Members of the International Scleroderma Group, Members of the Type 1 Diabetes Genetics Consortium (T1DGC). (PDF 1590 kb) [file 13073_2018_604_MOESM2_ESM.pdf]

Supplemental Material

Supplemental Tables ……………………………………………………………….......2

Supplemental Figures ...……………………………………………………….........6

Supplemental Note ………………...……………………………………………….15

Members of the Coeliac Disease Immunochip Consortium ………………….............15

Members of the RACI ……………………………………………………………18

Members of the International Scleroderma Group …………………………………….22

Members of the Type 1 Diabetes Genetics Consortium (T1DGC) ……………...….....25

Supplemental Tables

Table S3. Results of the subset-based meta-analysis for the lead variants showing evidence of opposite allelic effect across the autoimmune diseases contributing to the association signal.

Region	Position (bp)	SNP	Gene	A1	P2sided	Pheno Risk	P-value risk	OR [95% CI] risk	Pheno Protective	P-value protective	OR [95% CI] Potective
2q31.3	182,057,640	rs12619531	*ITGA4*	G	1.18E-18	CeD	1.65E-18	1.18 [1.13-1.22]	**SSc**	1.55E-02	0.92 [0.86-0.98]
3q25.33	159,647,674	rs17753641	*IL12A*	G	1.64E-29	CeD	2.69E-28	1.38 [1.30-1.46]	**SSc**	8.49E-04	0.80 [0.71-0.91]
5q33.1	150,438,988	rs1422673	*TNIP1*	T	1.87E-09	**RA** SSc	3.37E-09	1.11 [1.07-1.15]	**CeD**	2.28E-02	0.94 [0.89-0.99]
6q25.3	159,470,417	rs212407	*TAGAP*	G	6.74E-14	CeD	1.26E-11	1.13 [1.09-1.18]	RA T1D	1.54E-04	0.95 [0.92-0.97]
7p12.1	51,015,193	rs7780389	*COBL*	T	2.25E-08	**RA**	4.75E-02	1.10 [1.00-1.20]	T1D	2.18E-08	0.71 [0.63-0.80]
10p15.1	6,116,254	rs72776098	*IL2RA*	A	7.10E-10	**SSc**	1.39E-03	1.50 [1.17-1.93]	T1D	2.02E-08	0.61 [0.52-0.73]
10q22.3	81,045,280	rs1250568	*ZMIZ1*	C	3.87E-15	**SSc T1D**	9.04E-03	1.06 [1.01-1.10]	CeD	1.13E-14	0.87 [0.84-0.90]
11q23.3	118,726,843	rs10892299	*DDX6*	T	2.25E-13	**T1D**	2.49E-02	1.07 [1.01-1.13]	CeD **SSc**	2.68E-13	0.87 [0.84-0.90]
12q13.2	56,470,625	rs11171739	*IKZF4*	C	1.87E-20	T1D	1.04E-18	1.23 [1.17-1.28]	RA	3.59E-04	0.93 [0.90-0.97]
15q25.1	79,234,957	rs34593439	*CTSH*	A	1.47E-14	CeD	2.21E-06	1.15 [1.08-1.21]	T1D	1.82E-10	0.77 [0.71-0.83]

Position (bp), base pair position in hg19; **SNP**, single nucleotide polymorphism**; Gene**, annotated gene as described in online methods; **A1**, alternative allele used in the logistic regression; **P2sided,** p-value from the two-sided subset-based meta-analysis; **Pheno risk**, risk (OR>1) disease subset from the subset-based meta-analysis; **Pheno protective**, protective (OR<1) disease subset from the subset-based meta-analysis. Diseases included in the best subset and for which identified associations have not been previously reported are shown in bold

Table S6. Coding variants in tight linkage disequilibrium ($r^2 \geq 0.8$) with lead non-coding polymorphisms according to the European population of the 1000 Genomes Project. All genetic variants were annotated using the variant effect predictor (VEP).

			**VEP annotation lead SNP**			**VEP annotation proxy**		
**Region**	**Position (bp)**	**Lead SNP**	**Consequence**	**Gene symbol**	**Proxy SNP**	**Gene symbol**	**Consequence**	**SIFT**
1p36.32	2,534,978	rs6664969	Intron	*MMEL1*	rs3748816	*MMEL1*	Missense	Tolerated
					rs4648562	*MMEL1*	Synonymous	-
					rs4648658	*MMEL1*	Synonymous	-
					rs4648659	*MMEL1*	Synonymous	-
1p36.13	17,655,407	rs1748041	Intron	*PADI4*	rs1748033	*PADI4*	Synonymous	-
1q32.1	200,875,242	rs12132349	Intron	*C1orf106*	rs296520	*C1orf106*	Missense	Tolerated
7q32.1	128,572,766	rs4731532	Upstream	*IRF5*	rs2004640	*IRF5*	Splice donor	-
11p15.5	2,163,110	rs4244808	Intron	*INS-IGF2*	rs10770125	*IGF2*	Missense	Deleterious
15q25.1	79,234,957	rs34593439	Intron	*CTSH*	rs2289702	*CTSH*	Missense	Deleterious
16p11.2	28,505,660	rs151234	Upstream	*CLN3*	rs151233	*CLN3*	Synonymous	-
16q23.1	75,247,391	rs7202844	Intergenic	-	rs2287990	*CTRB1*	Synonymous	-
22q11.1	21,936,152	rs66534072	Intron	*UBE2L3*	rs2298428	*YJDC*	Missense	Tolerated
22q12.3	37,587,111	rs229533	Upstream	*C1QTNF6*	rs229527	*C1QTNF6*	Missense	Tolerated

Position (bp), base pair position in hg19; **SNP**, single nucleotide polymorphism; **Consequence**: consequence type of this variation on the protein sequence; **Gene symbol**, affected gene; **SIFT**, SIFT prediction if available.

Table S9. Transcription factor binding sites (TFBSs) potentially disrupted by the set of pleiotropic variants. The GenomeRunner web server was used to calculate enrichment p-values using Chi-squared test by evaluating whether the set of common SNPs co-localizes with TFBSs from the ENCODE project more often than expected by chance (see Methods).

TF	P-value	P_{FDR}	Direction
BATF	3.98E-17	6.40E-15	Over
RELA	7.59E-14	6.11E-12	Over
IRF4	3.50E-10	1.88E-08	Over
RUNX3	9.68E-09	3.89E-07	Over
SPI1	8.86E-08	2.38E-06	Over
MTA3	7.41E-08	2.38E-06	Over
POU2F2	1.05E-05	2.41E-04	Over
ATF2	9.72E-05	1.96E-03	Over
ELF1	2.41E-04	4.08E-03	Over
IKZF1	2.53E-04	4.08E-03	Over
USF2	4.17E-04	6.10E-03	Over
TAF1	1.79E-03	2.40E-02	Over

TF, transcription factor; $\mathbf{P_{FDR}}$, p-value after multiple testing correction using the Benjamini–Hochberg false discovery rate (FDR) procedure; **Direction**, direction of the enrichment. Over denotes overrepresentation of regulatory elements.

Table S10. Biological pathways significantly enriched among the set of common genes. KEGG pathway analysis was performed using WebGestalt (WEB-based GEne SeT AnaLysis Toolkit) with the human genome as the reference set, the Benjamini Hochberg adjustment for multiple testing, and a minimum number of 2 genes per category.

KEEG pathway	Genes	Enrichment statistics
Th1 and Th2 cell differentiation	*GATA3, RBPJ, IL2RA, IL12A, PRKCQ, STAT4, TYK2*	C=92; O=7; E=0.24; R=28.73; P-value=2.01e-09; FDR=6.21e-07
Measles	*IL2RA, FASLG, IL12A, PRKCQ, TNFAIP3, TYK2, CD28*	C=136; O=7; E=0.36; R=19.44; P-Value=3.14e-08; FDR=4.85e-06
Jak-STAT signaling pathway	*IL2RA, IL12A, PTPN2, STAT4, TYK2*	C=158; O=5; E=0.42; R=11.95; P-value=4.4e-05; FDR=4.53e-03
Allograft rejection	*FASLG, IL12A, CD28*	C=38; O=3; E=0.1; R=29.81; P-value=1.25e-04; FDR=7.85e-03
T cell receptor signaling pathway	*RASGRP1, CTLA4, PRKCQ, CD28*	C=105; O=4; E=0.28; R=14.39; P-value=1.42e-04; FDR=7.85e-03
Th17 cell differentiation	*GATA3, IL2RA, PRKCQ, TYK2*	C=107; O=4; E=0.28; R=14.12; P-value=1.53e-04; FDR=7.85e-03
Type I diabetes mellitus	*FASLG, IL12A, CD28*	C=43; O=3; E=0.11; R=26.35; P-value=1.82e-04; FDR=8.02e-03
Autoimmune thyroid disease	*CTLA4, FASLG, CD28*	C=53; O=3; E=0.14; R=21.38; P-value=3.39e-04; FDR=1.31e-02
Inflammatory bowel disease (IBD)	*GATA3, IL12A, STAT4*	C=65; O=3; E=0.17; R=17.43; P-value=6.2e-04; FDR=2.13e-02

C, the number of reference genes in the category; **O**, the number of genes in the user gene list and also in the category; **E**, the expected number in the category; **R**, ratio of enrichment; **P-value**, p value from hypergeometric test; **FDR**, p-value after multiple testing correction using the Benjamini–Hochberg false discovery rate (FDR) procedure.

Supplemental Figures

Figure S1. Quantile–quantile plots for the p-values of each individual disease, celiac disease (a), rheumatoid arthritis (b), systemic sclerosis (c), and type 1 diabetes (d), and the cross disease meta-analysis (e). The x-axis indicates the expected distribution of -log10 (P values) and the y-axis indicates the observed distribution of -log10 (P values). Genomic inflation of the observed distribution is indicated by λ and λ_{1000}.

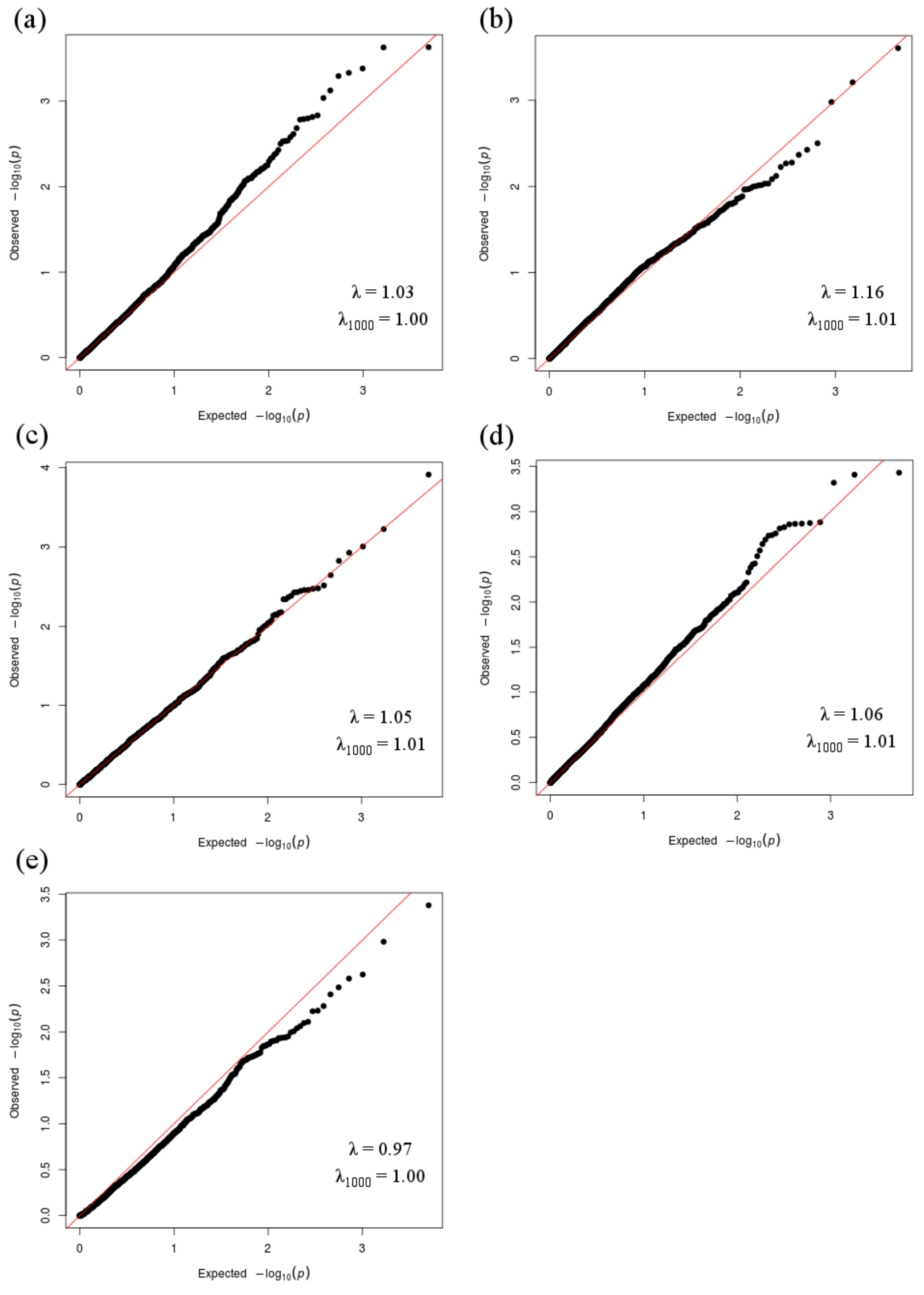

Figure S2. Empirical −log10(P)-distribution of the Zmax statistic obtained by simulating 300 × 10^6 replicates of four normally distributed random variables.

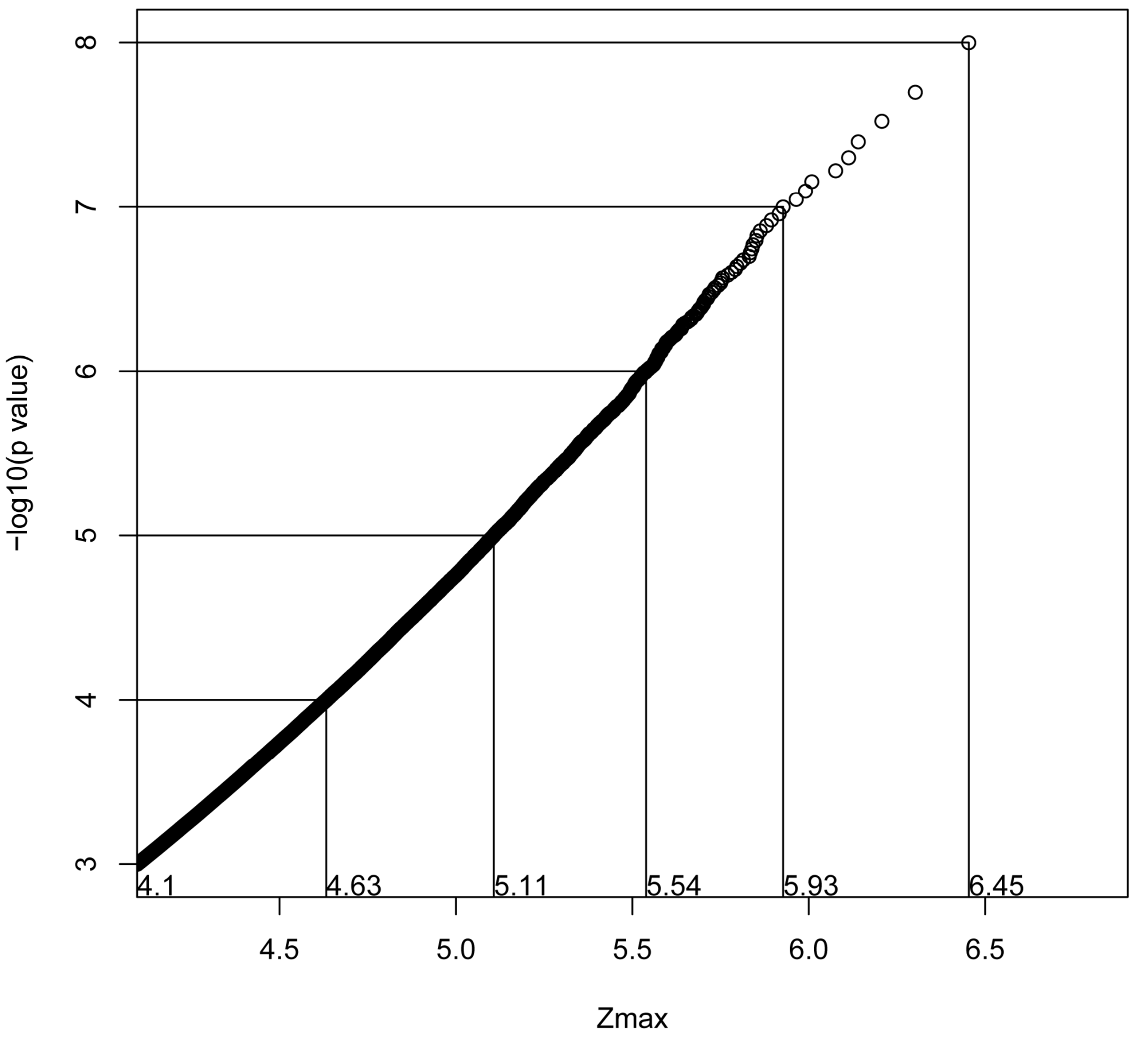

Figure S3. Manhattan plot of the subset-based meta-analysis of Immunochip data from celiac disease (CeD), systemic sclerosis (SSc), rheumatoid arthritis (RA) and type 1 diabetes (T1D). After applying quality control filters and imputation (see methods), we analyzed 252,970 polymorphisms in 37,159 autoimmune-disease patients and 22,308 healthy controls. The -log10 of the subset-based meta-analysis p-values are plotted against their physical chromosomal position. The red line represents the genome-wide level of significance ($p = 5x10^{-8}$).

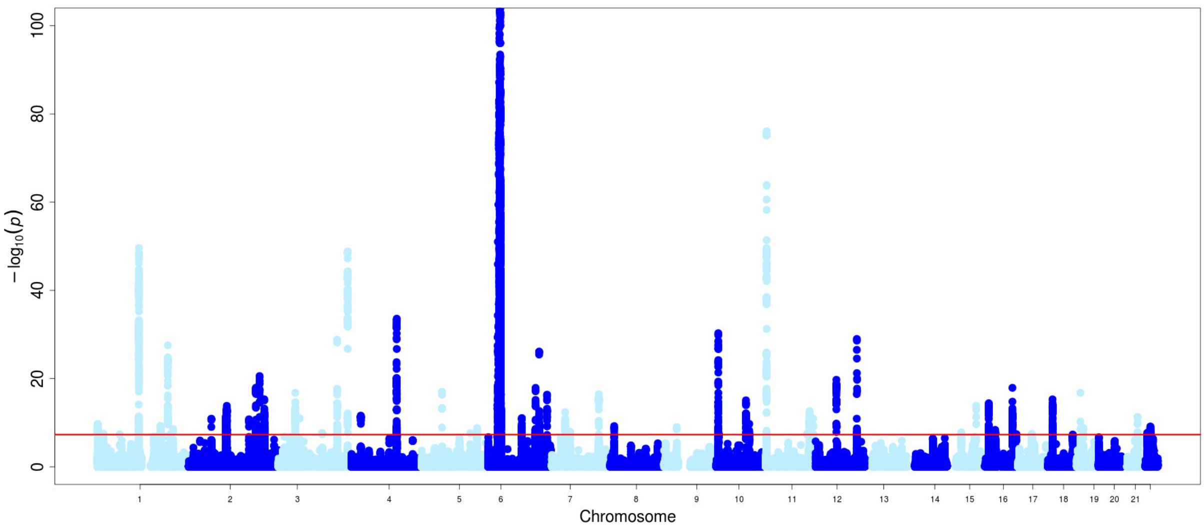

Figure S4. Disease-specific odds ratio for the pleiotropic variants showing opposite allelic effects across autoimmune diseases. Circles represent the analyzed diseases (red: celiac disease; yellow: rheumatoid arthritis; green: systemic sclerosis; blue: type 1 diabetes). Large circles denote diseases contributing to the association signal (i.e. included in the best subset).

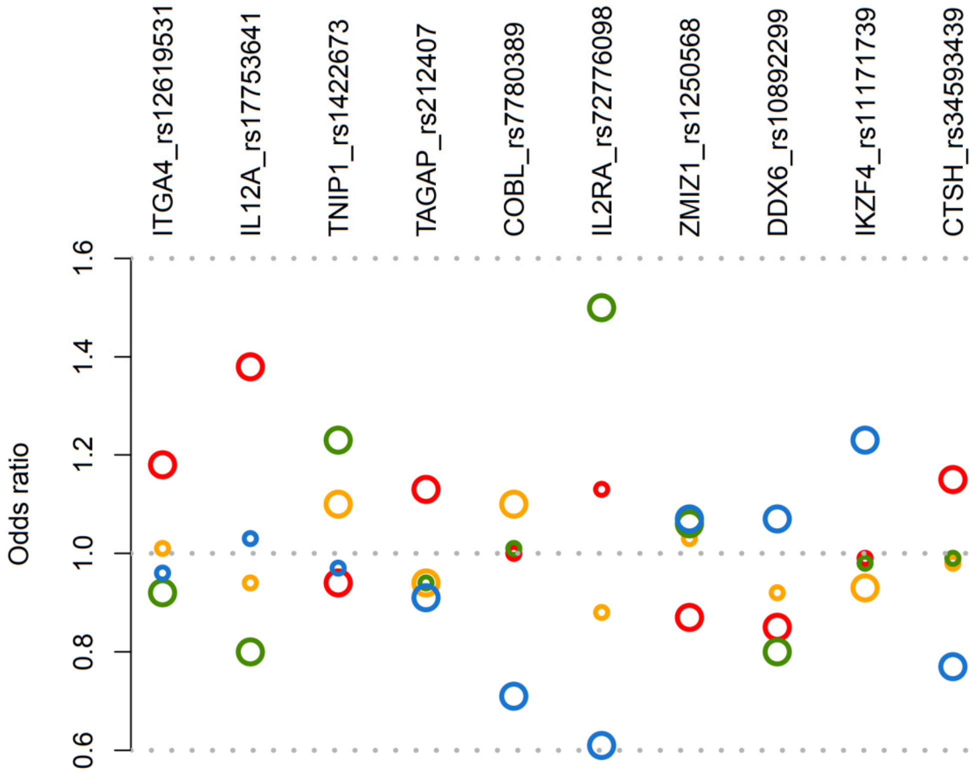

Figure S5. Regional association plots of the novel genome-wide associated locus for rheumatoid arthritis (RA), 2q32.3. (a) Results of the RA meta-analysis for the 2q32.3 region. Negative log10-tranformed p-value is shown for SNPs in the regions flanking 500 kb on either side of the index SNP (purple diamond). Both the most significance SNP within the region (index SNP) and the new genome-wide associated variant (rs10931468) are indicated in the plot. The index SNP is shown as a diamond and the r^2 values of the remaining SNPs are indicated by color (see legend). (b) Results of the RA meta-analysis for the 2q32.3 region after conditioning for the most associated SNP (rs7582694). The genes within the region are annotated and indicated by arrows. Plots were generated using LocusZoom (http://locuszoom.org/).

(a)

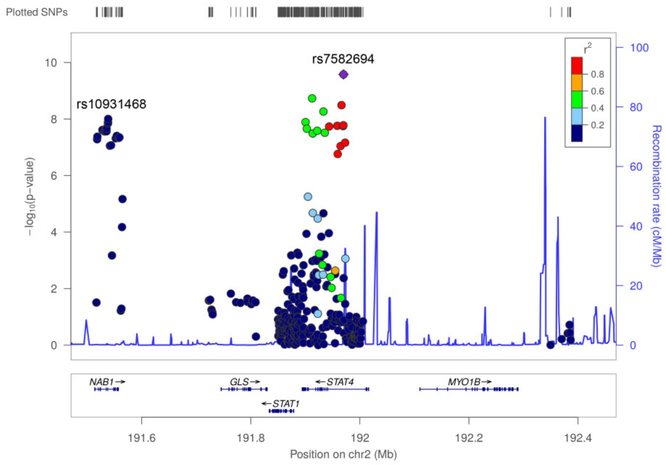

(b)

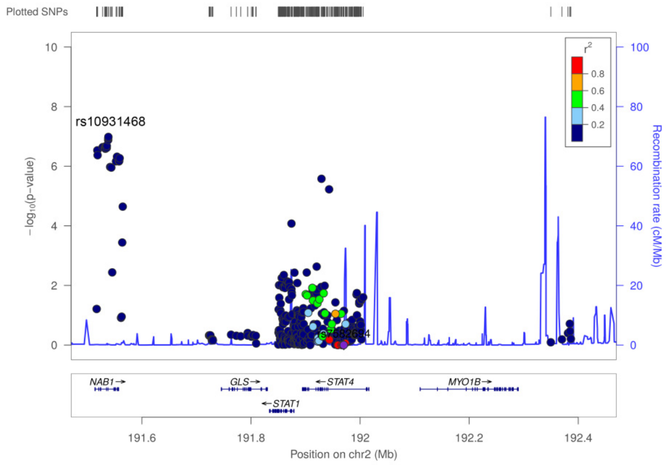

Figure S6. Regional association plots of the novel genome-wide associated loci for systemic sclerosis (SSc), 11q23.3 (a), 1q25.1 (b), and 1q25.3 (c). Results of the SSc meta-analysis (−log10 P) are shown for SNPs in the regions flanking 500 kb on either side of the index SNPs (purple diamonds). The index SNPs are shown as diamonds and the r^2 values of the remaining SNPs are indicated by color (see legend). The genes within the region are annotated and indicated by arrows. Plots were generated using LocusZoom (http://locuszoom.org/).

(a)

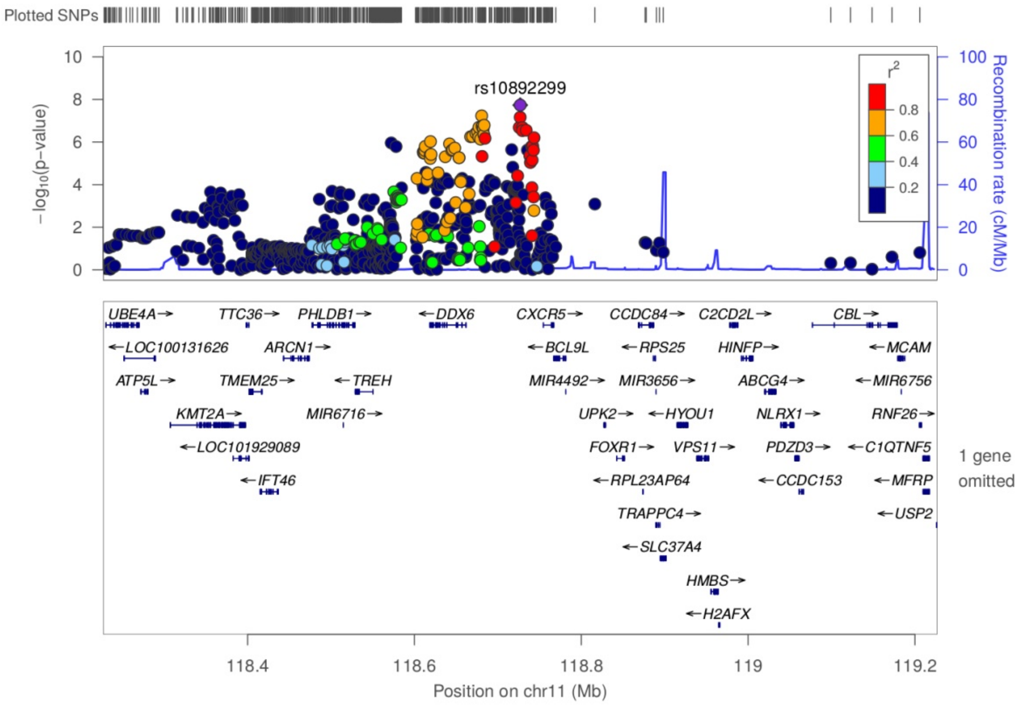

(b)

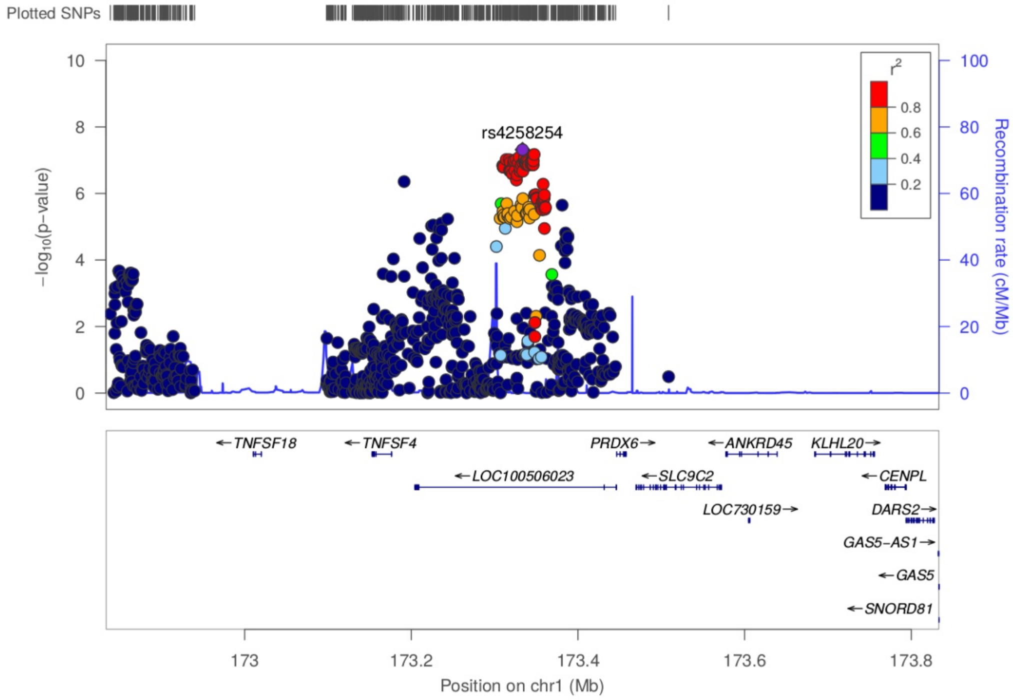
Plotted SNPs
rs4258254
−log10(p−value)
Recombination rate (cM/Mb)
r2
0.8
0.6
0.4
0.2
0
2
4
6
8
10
0
20
40
60
80
100
←TNFSF18
←TNFSF4
PRDX6→
←ANKRD45
KLHL20→
←LOC100506023
←SLC9C2
←CENPL
LOC730159→
DARS2→
GAS5−AS1→
←GAS5
←SNORD81
173
173.2
173.4
173.6
173.8
Position on chr1 (Mb)

(c)

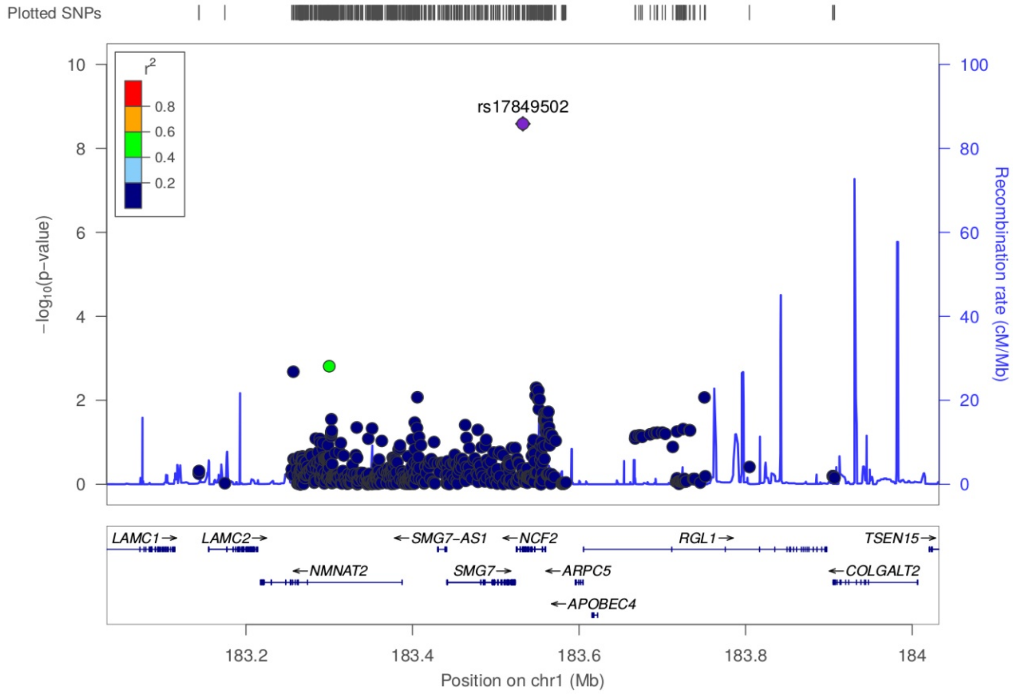
Plotted SNPs
rs17849502
−log10(p−value)
Recombination rate (cM/Mb)
r2
0.8
0.6
0.4
0.2
0
2
4
6
8
10
0
20
40
60
80
100
LAMC1→
LAMC2→
←SMG7−AS1
←NCF2
RGL1→
TSEN15→
←NMNAT2
SMG7→
←ARPC5
←COLGALT2
←APOBEC4
183.2
183.4
183.6
183.8
184
Position on chr1 (Mb)

Figure S7. Regional association plot of the novel genome-wide associated locus for celiac disease (CeD), 3p14.1. Results of the CeD meta-analysis (−log10 P) are shown for SNPs in the region flanking 500 kb on either side of the index SNP. The index SNP is shown as a diamond and the r^2 values of the remaining SNPs are indicated by color (see legend). The genes within the region are annotated and indicated by arrows. Plot was generated using LocusZoom (http://locuszoom.org/).

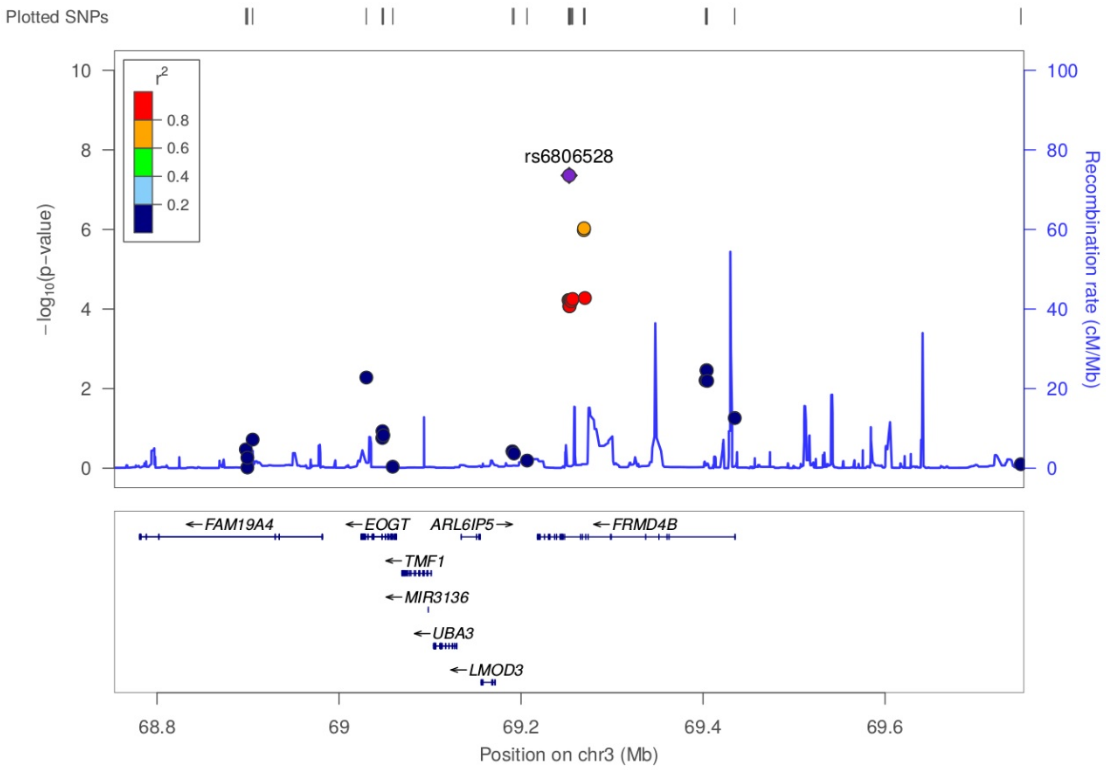

Figure S8. Regional association plot of the novel genome-wide associated locus for type 1 diabetes (T1D), 16p12.3. Results of the T1D analysis (−log10 P) are shown for SNPs in the regions flanking 500 kb on either side of the index SNP (purple diamond). The index SNP is shown as a diamond and the r^2 values of the remaining SNPs are indicated by color (see legend). The genes within the region are annotated and indicated by arrows. Plots were generated using LocusZoom (http://locuszoom.org/).

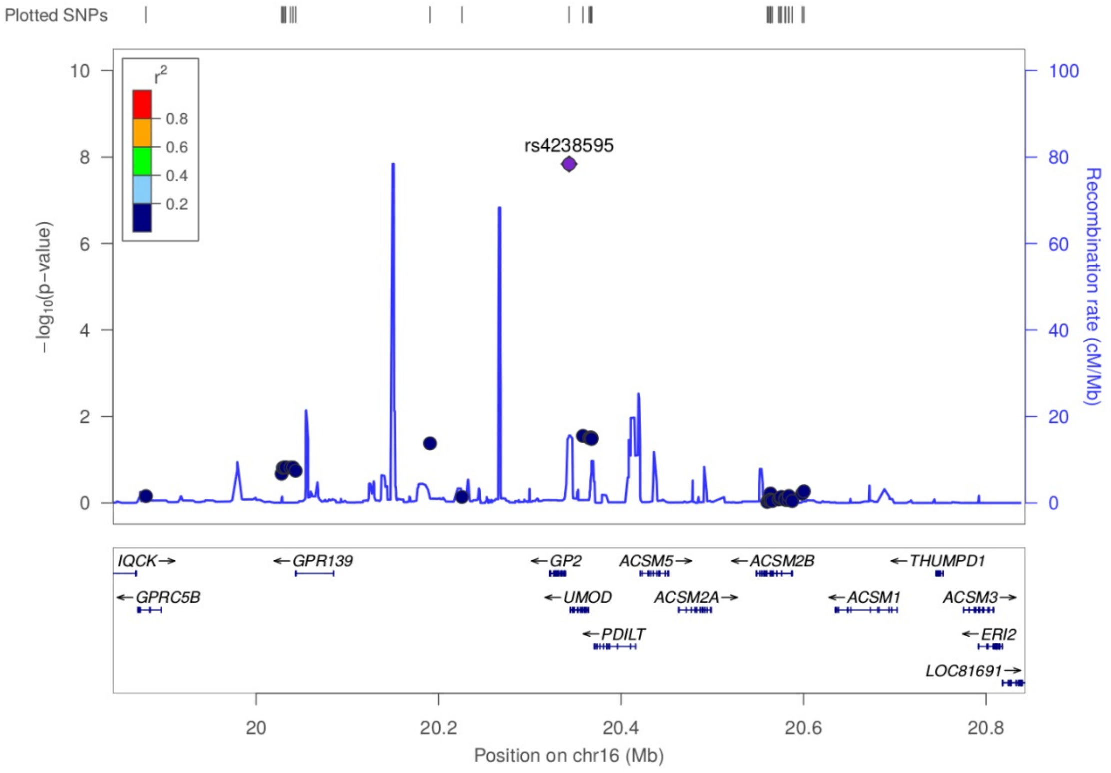

Supplemental Note

Members of the Coeliac Disease Immunochip Consortium

Gosia Trynka (University Medical Center and University of Groningen, Groningen, The Netherlands); Karen A Hunt (Blizard Institute, Barts and The London School of Medicine and Dentistry, Queen Mary University of London, London, United Kingdom); Nicholas A Bockett (Blizard Institute, Barts and The London School of Medicine and Dentistry, Queen Mary University of London, London, United Kingdom); Jihane Romanos (University Medical Center and University of Groningen, Groningen, The Netherlands); Vanisha Mistry (Blizard Institute, Barts and The London School of Medicine and Dentistry, Queen Mary University of London, London, United Kingdom); Agata Szperl (University Medical Center and University of Groningen, Groningen, The Netherlands); Sjoerd F Bakker (VU Medical Center, Amsterdam, The Netherlands); Maria Teresa Bardella (Fondazione IRCCS Ospedale Maggiore Policlinico, Mangiagalli e Regina Elena, Milan, Italy; Department of Medical Sciences, University of Milan, Milan, Italy); Leena Bhaw-Rosun (Genome Centre, Barts and the London School of Medicine and Dentistry, John Vane Science Centre, London, United Kingdom); Gemma Castillejo (Universitat Rovira I Virgili, Hospital Univesitari de Sant Joan de Reus, Reus, Spain); Emilio G. de la Concha (Hospital Clínico S. Carlos, Instituto de Investigación Sanitaria San Carlos IdISSC, Madrid, Spain); Rodrigo Coutinho de Almeida (University Medical Center and University of Groningen, Groningen, The Netherlands); Kerith-Rae M Dias (Genome Centre, Barts and the London School of Medicine and Dentistry, John Vane Science Centre, London, United Kingdom); Cleo C. van Diemen (University Medical Center and University of Groningen, Groningen, The Netherlands); Patrick CA Dubois (Blizard Institute, Barts and The London School of Medicine and Dentistry, Queen Mary University of London, London, United Kingdom); Richard H. Duerr (Department of Medicine, University of Pittsburgh School of Medicine, Pittsburgh, Pennsylvania, USA; Department of Human Genetics, University of Pittsburgh Graduate School of Public Health, Pittsburgh, Pennsylvania, USA); Sarah Edkins (Wellcome Trust Sanger Institute, Hinxton, Cambridge, United Kingdom); Lude Franke (University Medical Center and University of Groningen, Groningen, The Netherlands); Karin Fransen (University Medical Center and University of Groningen, Groningen, The Netherlands); Javier Gutierrez (University Medical Center and University of Groningen,

Groningen, The Netherlands); Graham AR Heap (Blizard Institute, Barts and The London School of Medicine and Dentistry, Queen Mary University of London, London, United Kingdom); Barbara Hrdlickova (University Medical Center and University of Groningen, Groningen, The Netherlands); Sarah Hunt (Wellcome Trust Sanger Institute, Hinxton, Cambridge, United Kingdom); Leticia Plaza Izurieta (Immunogenetics Research Laboratory, Hospital de Cruces, Barakaldo, Bizkaia, Spain); Valentina Izzo (European Laboratory for Food Induced Disease, University of Naples Federico II, Naples, Italy); Leo AB Joosten (Department of Medicine, Radboud University Nijmegen Medical Centre, Nijmegen, The Netherlands; Nijmegen Institute for Infection, Inflammation and Immunity (N4i), Radboud University Nijmegen Medical Centre, Nijmegen, The Netherlands); Cordelia Langford (Wellcome Trust Sanger Institute, Hinxton, Cambridge, United Kingdom); Maria Cristina Mazzilli (Sapienza University of Rome, Rome, Italy); Charles A Mein (Genome Centre, Barts and the London School of Medicine and Dentistry, John Vane Science Centre, London, United Kingdom); Vandana Midah (Dayanand Medical College and Hospital, Ludhiana, Punjab, India); Mitja Mitrovic (University Medical Center and University of Groningen, Groningen, The Netherlands; University of Maribor, Center for Human Molecular Genetics and Pharmacogenomics, Maribor, Slovenia); Barbara Mora (Sapienza University of Rome, Rome, Italy); Marinita Morelli (European Laboratory for Food Induced Disease, University of Naples Federico II, Naples, Italy); Sarah Nutland (Juvenile Diabetes Research Foundation/Wellcome Trust Diabetes and Inflammation Laboratory, Cambridge Institute for Medical Research, University of Cambridge, Cambridge, United Kingdom); Concepción Núñez (Hospital Clínico S. Carlos, Instituto de Investigación Sanitaria San Carlos IdISSC, Madrid, Spain); Suna Onengut-Gumuscu (Center for Public Health Genomics, University of Virginia, Charlottesville, VA, USA); Kerra Pearce (UCL Genomics, Institute of Child Health, University College London, London, United Kingdom); Mathieu Platteel (University Medical Center and University of Groningen, Groningen, The Netherlands); Isabel Polanco (Hospital La Paz, Madrid, Spain); Simon Potter (Wellcome Trust Sanger Institute, Hinxton, Cambridge, United Kingdom); Carmen Ribes-Koninckx (La Fe University Hospital, Valencia, Spain); Isis Ricaño-Ponce (University Medical Center and University of Groningen, Groningen, The Netherlands); Stephen S. Rich (Center for Public Health Genomics, University of Virginia, Charlottesville, VA, USA); Anna Rybak (Children’s Memorial Health Institute, Warsaw, Poland); José Luis Santiago (Hospital Clínico S. Carlos, Instituto de

Investigación Sanitaria San Carlos IdISSC, Madrid, Spain); Sabyasachi Senapati (University of Delhi, South Campus, New Delhi, India); Ajit Sood (Dayanand Medical College and Hospital, Ludhiana, Punjab, India); Hania Szajewska (The Medical University of Warsaw, Warsaw, Poland); Riccardo Troncone (University of Naples, Fedrico II, Naples, Italy); Jezabel Varadé (Hospital Clínico S. Carlos, Instituto de Investigación Sanitaria San Carlos IdISSC, Madrid, Spain); Chris Wallace (Juvenile Diabetes Research Foundation/Wellcome Trust Diabetes and Inflammation Laboratory, Cambridge Institute for Medical Research, University of Cambridge, United Kingdom); Victorien M Wolters (University Medical Centre Utrecht, Utrecht, The Netherlands); Alexandra Zhernakova (Leiden University Medical Center, Leiden, The Netherlands); CEGEC (Spanish Consortium on the Genetics of Coeliac Disease), PreventCD Study Group, Wellcome Trust Case Control Consortium, B.K. Thelma (University of Delhi, South Campus, New Delhi, India); Bozena Cukrowska (Children's Memorial Health Institute, Warsaw, Poland); Elena Urcelay (Hospital Clínico S. Carlos, Instituto de Investigación Sanitaria San Carlos IdISSC, Madrid, Spain); Jose Ramon Bilbao (Immunogenetics Research Laboratory, Hospital de Cruces, Barakaldo, Bizkaia, Spain); M Luisa Mearin (Leiden University Medical Centre, Leiden, The Netherlands); Donatella Barisani (Faculty of Medicine University of Milano-Bicocca,Monza, Italy); Jeffrey C Barrett (Wellcome Trust Sanger Institute, Hinxton, Cambridge, United Kingdom); Vincent Plagnol (UCL Genetics Institute, University College London, London); Panos Deloukas (Wellcome Trust Sanger Institute, Hinxton, Cambridge, United Kingdom); Cisca Wijmenga (University Medical Center and University of Groningen, Groningen, The Netherlands); David A van Heel (Blizard Institute, Barts and The London School of Medicine and Dentistry, Queen Mary University of London, London, United Kingdom).

Members of the RACI

Steve Eyre (Arthritis Research UK Epidemiology Unit, Centre for Musculoskeletal Research, University of Manchester, Manchester Academic Health Science Centre, Manchester, UK; National Institute for Health Research, Manchester Musculoskeletal Biomedical Research Unit, Central Manchester University Hospitals National Health Service Foundation Trust, Manchester Academic Health Sciences Centre, Manchester, UK); John Bowes (Arthritis Research UK Epidemiology Unit, Centre for Musculoskeletal Research, University of Manchester, Manchester Academic Health Science Centre, Manchester, UK; National Institute for Health Research, Manchester Musculoskeletal Biomedical Research Unit, Central Manchester University Hospitals National Health Service Foundation Trust, Manchester Academic Health Sciences Centre, Manchester, UK); Dorothée Diogo (Division of Rheumatology, Immunology and Allergy, Brigham and Women's Hospital, Harvard Medical School, Boston, Massachusetts, USA; Division of Genetics, Brigham and Women's Hospital, Harvard Medical School, Boston, Massachusetts, USA; Program in Medical and Population Genetics, Broad Institute, Cambridge, Massachusetts, USA); Annette Lee (The Feinstein Institute for Medical Research, North Shore–Long Island Jewish Health System, Manhasset, New York, USA); Anne Barton (Arthritis Research UK Epidemiology Unit, Centre for Musculoskeletal Research, University of Manchester, Manchester Academic Health Science Centre, Manchester, UK; National Institute for Health Research, Manchester Musculoskeletal Biomedical Research Unit, Central Manchester University Hospitals National Health Service Foundation Trust, Manchester Academic Health Sciences Centre, Manchester, UK); Paul Martin (Arthritis Research UK Epidemiology Unit, Centre for Musculoskeletal Research, University of Manchester, Manchester Academic Health Science Centre, Manchester, UK; National Institute for Health Research, Manchester Musculoskeletal Biomedical Research Unit, Central Manchester University Hospitals National Health Service Foundation Trust, Manchester Academic Health Sciences Centre, Manchester, UK); Alexandra Zhernakova (Department of Rheumatology, Leiden University Medical Centre, Leiden, The Netherlands; Department of Genetics, University Medical Center Groningen and University of Groningen, Groningen, The Netherlands); Eli Stahl (Division of Rheumatology, Immunology and Allergy, Brigham and Women's Hospital, Harvard Medical School, Boston, Massachusetts, USA; Division of Genetics, Brigham and Women's Hospital, Harvard Medical School, Boston, Massachusetts, USA; Program in

Medical and Population Genetics, Broad Institute,Cambridge, Massachusetts, USA); Sebastien Viatte (Arthritis Research UK Epidemiology Unit, Centre for Musculoskeletal Research, University of Manchester, Manchester Academic Health Science Centre, Manchester, UK; National Institute for Health Research, Manchester Musculoskeletal Biomedical Research Unit, Central Manchester University Hospitals National Health Service Foundation Trust, Manchester Academic Health Sciences Centre, Manchester, UK); Kate McAllister (Arthritis Research UK Epidemiology Unit, Centre for Musculoskeletal Research, University of Manchester, Manchester Academic Health Science Centre, Manchester, UK; National Institute for Health Research, Manchester Musculoskeletal Biomedical Research Unit, Central Manchester University Hospitals National Health Service Foundation Trust, Manchester Academic Health Sciences Centre, Manchester, UK); Christopher I Amos (University of Texas MD Anderson Cancer Center, Houston, Texas, USA); Leonid Padyukov (Rheumatology Unit, Department of Medicine, Karolinska Institutet and Karolinska University Hospital Solna, Stockholm, Sweden); Rene E M Toes (Department of Rheumatology, Leiden University Medical Centre, Leiden, The Netherlands); Tom W J Huizinga (Department of Rheumatology, Leiden University Medical Centre, Leiden, The Netherlands); Cisca Wijmenga (Department of Genetics, University Medical Center Groningen and University of Groningen, Groningen, The Netherlands); Gosia Trynka (Division of Rheumatology, Immunology and Allergy, Brigham and Women's Hospital, Harvard Medical School, Boston, Massachusetts, USA; Division of Genetics, Brigham and Women's Hospital, Harvard Medical School, Boston, Massachusetts, USA; Program in Medical and Population Genetics, Broad Institute,Cambridge, Massachusetts, USA; Department of Genetics, University Medical Center Groningen and University of Groningen, Groningen, The Netherlands); Lude Franke (Department of Genetics, University Medical Center Groningen and University of Groningen, Groningen, The Netherlands); Harm-Jan Westra (Department of Genetics, University Medical Center Groningen and University of Groningen, Groningen, The Netherlands): Lars Alfredsson (Department of Environmental Medicine, Karolinska Institutet, Stockholm, Sweden); Xinli Hu (Division of Rheumatology, Immunology and Allergy, Brigham and Women's Hospital, Harvard Medical School, Boston, Massachusetts, USA; Division of Genetics, Brigham and Women's Hospital, Harvard Medical School, Boston, Massachusetts, USA; Program in Medical and Population Genetics, Broad Institute, Cambridge, Massachusetts, USA; Harvard–Massachusetts Institute of Technology Division of

Health Sciences and Technology, Boston, Massachusetts, USA); Cynthia Sandor (Division of Rheumatology, Immunology and Allergy, Brigham and Women's Hospital, Harvard Medical School, Boston, Massachusetts, USA; Division of Genetics, Brigham and Women's Hospital, Harvard Medical School, Boston, Massachusetts, USA; Program in Medical and Population Genetics, Broad Institute,Cambridge, Massachusetts, USA); Paul I W de Bakker (Division of Rheumatology, Immunology and Allergy, Brigham and Women's Hospital, Harvard Medical School, Boston, Massachusetts, USA; Division of Genetics, Brigham and Women's Hospital, Harvard Medical School, Boston, Massachusetts, USA; Program in Medical and Population Genetics, Broad Institute,Cambridge, Massachusetts, USA; Department of Epidemiology, University Medical Center Utrecht, Utrecht, The Netherlands; Department of Medical Genetics, University Medical Center Utrecht, Utrecht, The Netherlands); Sonia Davila (Division of Human Genetics, Genome Institute of Singapore, Singapore); Chiea Chuen Khor (Division of Human Genetics, Genome Institute of Singapore, Singapore); Khai Koon Heng (Division of Human Genetics, Genome Institute of Singapore, Singapore); Robert Andrews (The Wellcome Trust Sanger Institute, Cambridge, UK); Sarah Edkins (The Wellcome Trust Sanger Institute, Cambridge, UK); Sarah E Hunt (The Wellcome Trust Sanger Institute, Cambridge, UK); Cordelia Langford (The Wellcome Trust Sanger Institute, Cambridge, UK); Deborah Symmons (Arthritis Research UK Epidemiology Unit, Centre for Musculoskeletal Research, University of Manchester, Manchester Academic Health Science Centre, Manchester, UK; National Institute for Health Research, Manchester Musculoskeletal Biomedical Research Unit, Central Manchester University Hospitals National Health Service Foundation Trust, Manchester Academic Health Sciences Centre, Manchester, UK); Biologics in Rheumatoid Arthritis Genetics and Genomics Study Syndicate; Wellcome Trust Case Control Consortium; Pat Concannon (Center for Public Health Genomics, University of Virginia, Charlottesville, Virginia, USA); Suna Onengut-Gumuscu (Center for Public Health Genomics, University of Virginia, Charlottesville, Virginia, USA); Stephen S Rich (Center for Public Health Genomics, University of Virginia, Charlottesville, Virginia, USA); Panos Deloukas (The Wellcome Trust Sanger Institute, Cambridge, UK); Miguel A Gonzalez-Gay (Department of Rheumatology, Hospital Marques de Valdecilla, Instituto de Fundación e Investigación Marqués de Valdecilla, Santander, Spain); Luis Rodriguez-Rodriguez (Hospital Clinico San Carlos, Madrid, Spain); Lisbeth Ärlsetig (Department of Public Health and Clinical

Medicine, Umeå University, Umeå, Sweden; Department of Rheumatology, Umeå University, Umeå, Sweden); Javier Martin (Instituto de Parasitología y Biomedicina "López-Neyra", CSIC, PTS Granada, Granada, Spain); Solbritt Rantapää-Dahlqvist (Department of Public Health and Clinical Medicine, Umeå University, Umeå, Sweden; Department of Rheumatology, Umeå University, Umeå, Sweden); Robert M Plenge (Division of Rheumatology, Immunology and Allergy, Brigham and Women's Hospital, Harvard Medical School, Boston, Massachusetts, USA; Division of Genetics, Brigham and Women's Hospital, Harvard Medical School, Boston, Massachusetts, USA; Program in Medical and Population Genetics, Broad Institute,Cambridge, Massachusetts, USA); Soumya Raychaudhuri (Arthritis Research UK Epidemiology Unit, Centre for Musculoskeletal Research, University of Manchester, Manchester Academic Health Science Centre, Manchester, UK; National Institute for Health Research, Manchester Musculoskeletal Biomedical Research Unit, Central Manchester University Hospitals National Health Service Foundation Trust, Manchester Academic Health Sciences Centre, Manchester, UK; Division of Rheumatology, Immunology and Allergy, Brigham and Women's Hospital, Harvard Medical School, Boston, Massachusetts, USA; Division of Genetics, Brigham and Women's Hospital, Harvard Medical School, Boston, Massachusetts, USA; Program in Medical and Population Genetics, Broad Institute,Cambridge, Massachusetts, USA); Lars Klareskog (Rheumatology Unit, Department of Medicine, Karolinska Institutet and Karolinska University Hospital Solna, Stockholm, Sweden); Peter K Gregersen (The Feinstein Institute for Medical Research, North Shore–Long Island Jewish Health System, Manhasset, New York, USA); Jane Worthington (Arthritis Research UK Epidemiology Unit, Centre for Musculoskeletal Research, University of Manchester, Manchester Academic Health Science Centre, Manchester, UK; National Institute for Health Research, Manchester Musculoskeletal Biomedical Research Unit, Central Manchester University Hospitals National Health Service Foundation Trust, Manchester Academic Health Sciences Centre, Manchester, UK).

Members of the International Scleroderma Group

Maureen D. Mayes (The University of Texas Health Science Center at Houston, Houston, TX, USA); Lara Bossini-Castillo (Instituto de Parasitología y Biomedicina López-Neyra, Consejo Superior de Investigaciones Científicas, Granada, Spain); Olga Gorlova (The University of Texas MD Anderson Cancer Center, Houston, TX, USA); Xiaodong Zhou (The University of Texas Health Science Center at Houston, Houston, TX, USA); Wei V. Chen (The University of Texas MD Anderson Cancer Center, Houston, TX, USA); Shervin Assassi (The University of Texas Health Science Center at Houston, Houston, TX, USA); Jun Ying (The University of Texas MD Anderson Cancer Center, Houston, TX, USA); Filemon K. Tan (The University of Texas Health Science Center at Houston, Houston, TX, USA); Frank C. Arnett (The University of Texas Health Science Center at Houston, Houston, TX, USA); John D. Reveille (The University of Texas Health Science Center at Houston, Houston, TX, USA); Sandra Guerra (Centre for Rheumatology, University College London Medical School, London, UK); Peter K. Gregersen (The Feinstein Institute for Medical Research, North Shore – Long Island Jewish Health System, Manhasset, NY, USA); Annette T. Lee (The Feinstein Institute for Medical Research, North Shore – Long Island Jewish Health System, Manhasset, NY, USA); Carmen Pilar Simeón (Vall d'Hebron Hospital, Barcelona, Spain); Patricia Carreira (12 de Octubre University Hospital, Madrid, Spain); Iván Castellví (Santa Creu i Sant Pau University Hospital, Barcelona, Spain); Miguel A. González-Gay (Epidemiology, Genetics and Atherosclerosis Research Group on Systemic Inflammatory Diseases, IDIVAL, University of Cantabria, Santander, Spain); Lorenzo Beretta (Referral Center for Systemic Autoimmune Diseases, Fondazione IRCCS Ca' Granda Ospedale Maggiore Policlinico di Milano, Milan, Italy); Alexander E. Voskuyl (VU University Medical Center, Amsterdam, The Netherlands); Paolo Airò (ASST Spedali Civili, Brescia, Italy); Claudio Lunardi (Universita degli Studi di Verona, Verona, Italy); Paul Shiels (Glasgow Biomedical Research Centre, University of Glasgow, Glasgow, UK); Jacob M. van Laar (Institute of Cellular Medicine, Newcastle University, Newcastle upon Tyne, UK); Ariane Herrick (The University of Manchester, Manchester Academic Health Science Centre, Manchester, UK); Jane Worthington (The University of Manchester, Manchester Academic Health Science Centre, Manchester, UK); Christopher P. Denton (Royal Free and University College Medical School, London, UK); Jasper Broen (Radboud University Nijmegen Medical Centre, Nijmegen, the Netherlands; University Medical Center Utrecht,

Utrecht, the Netherlands); Timothy R.D.J. Radstake (Radboud University Nijmegen Medical Centre, Nijmegen, the Netherlands; University Medical Center Utrecht, Utrecht, the Netherlands); Carmen Fonseca (Centre for Rheumatology, University College London Medical School, London, UK); Bobby P. Koeleman (University Medical Center Utrecht, Utrecht, the Netherlands); Javier Martin (Instituto de Parasitología y Biomedicina López-Neyra, Consejo Superior de Investigaciones Científicas, Granada, Spain); Raquel Ríos (Hospital Clínico Universitario San Cecilio, Granada, Spain); Jose Luis Callejas, (Hospital Clínico Universitario San Cecilio, Granada, Spain); José Antonio Vargas Hitos (Hospital Virgen de las Nieves, Granada, Spain); Rosa García Portales (Hospital Virgen de la Victoria, Málaga, Spain); María Teresa Camps (Hospital Carlos Haya, Málaga, Spain); Antonio Fernández-Nebro (Hospital Carlos Haya, Málaga, Spain); María F. González-Escribano (Hospital Virgen del Rocío, Sevilla, Spain); Francisco José García-Hernández (Hospital Virgen del Rocío, Sevilla, Spain); Mª Jesús Castillo (Hospital Virgen del Rocío, Sevilla, Spain); Mª Ángeles Aguirre (Hospital Reina Sofía/IMIBIC, Córdoba, Spain); Inmaculada Gómez-Gracia (Hospital Reina Sofía/IMIBIC, Córdoba, Spain); Luis Rodríguez-Rodríguez (Hospital Clínico San Carlos, Madrid, Spain); Paloma García de la Peña (Madrid Norte Sanchinarro Hospital, Madrid, Spain); Esther Vicente (Hospital La Princesa, Madrid, Spain); José Luis Andreu (Hospital Puerta de Hierro Majadahonda, Madrid, Spain); Mónica Fernández de Castro (Hospital Puerta de Hierro Majadahonda, Madrid, Spain); Francisco Javier López-Longo (Hospital General Universitario Gregorio Marañón, Madrid, Spain); Lina Martínez (Hospital General Universitario Gregorio Marañón, Madrid, Spain); Vicente Fonollosa (Hospital Valle de Hebrón, Barcelona, Spain); Alfredo Guillén (Hospital Valle de Hebrón, Barcelona, Spain); Gerard Espinosa (Hospital Clinic, Barcelona, Spain); Carlos Tolosa (Hospital Parc Tauli, Sabadell, Spain); Anna Pros (Hospital Del Mar, Barcelona, Spain); Mónica Rodríguez Carballeira (Hospital Universitari Mútua Terrasa, Barcelona, Spain); Francisco Javier Narváez (Hospital Universitari de Bellvitge, Barcelona, Spain); Manel Rubio Rivas (Hospital Universitari de Bellvitge, Barcelona, Spain); Vera Ortiz-Santamaría (Hospital General de Granollers, Granollers, Spain); Ana Belén Madroñero (Hospital General San Jorge, Huesca, Spain); Bernardino Díaz (Hospital Central de Asturias, Oviedo, Spain); Luis Trapiella (Hospital Central de Asturias, Oviedo, Spain); Adrián Sousa (Hospital Xeral-Complexo Hospitalario Universitario de Vigo, Vigo, Spain); María Victoria Egurbide (Hospital Universitario Cruces, Barakaldo, Spain); Patricia Fanlo Mateo (Hospital

Virgen del Camino, Pamplona, Spain); Luis Sáez-Comet (Hospital Universitario Miguel Servet, Zaragoza, Spain); Federico Díaz (Hospital Universitario de Canarias, Tenerife, Spain); Vanesa Hernández (Hospital Universitario de Canarias, Tenerife, Spain); Emma Beltrán (Hospital General Universitario de Valencia, Valencia, Spain); José Andrés Román-Ivorra (Hospital Universitari i Politecnic La Fe, Valencia, Spain); Elena Grau (Hospital Universitari i Politecnic La Fe, Valencia, Spain); Juan José Alegre-Sancho (Hospital Universitari Doctor Peset, Valenci, Spain); Francisco J. Blanco García (INIBIC-Hospital Universitario A Coruña, La Coruña, Spain); Natividad Oreiro (INIBIC-Hospital Universitario A Coruña, La Coruña, Spain); Norberto Ortego-Centeno (Hospital Clínico Universitario San Cecilio, Granada, Spain); Mayka Freire (Complexo Hospitalario Universitario de Vigo, Vigo, Spain); Benjamín Fernández-Gutiérrez (Hospital Clínico San Carlos, Madrid, Spain); Alejandro Balsa (Hospital Universitario La Paz, Instituto de Investigación Sanitaria La Paz. IdiPAZ. Madrid, Spain); Ana M Ortiz (Hospital Universitario La Princesa, Instituto de Investigación Sanitaria La Princesa, Madrid, Spain); Jeska de Vries-Bouwstra (Leiden University Medical Center, Leiden, The Netherlands); Cesar Magro-Checa (Leiden University Medical Center, Leiden, The Netherlands); Alessandro Santaniello (Referral Center for Systemic Autoimmune Diseases, Fondazione IRCCS Ca' Granda Ospedale Maggiore Policlinico di Milano, Milan, Italy); Chiara Bellocchi (Referral Center for Systemic Autoimmune Diseases, Fondazione IRCCS Ca' Granda Ospedale Maggiore Policlinico di Milano, Milan, Italy); Gianluca Moroncini (Università Politecnica delle Marche and Ospedali Riuniti, Ancona, Italy); Armando Gabrielli (Università Politecnica delle Marche and Ospedali Riuniti, Ancona, Italy);

Members of the Type 1 Diabetes Genetics Consortium (T1DGC)

Asia---Pacific Network: Tracey Baskerville (Mater Children's Hospital, Australia); Nines Bautista (Institute for Study on Diabetes Foundation, Philippines); Eesh Bhatia (Sanjay Gandhi Postgraduate Institute, India); Vijayalakshmi Bhatia (Sanjay Gandhi Postgraduate Institute, India); Kamaruzaman Bin Hasan (National University of Malaysia Hospital, Malaysia); Francois Bonnici (University of Cape Town, South Africa); Thomas Brodnicki (Walter & Eliza Hall Institute of Medical Research, Australia); Brian Browning (The University of Auckland, New Zealand); Fergus Cameron (Royal Children's Hospital, Australia); Katharee Chaichanwatanakul (Mahidol University, Thailand); Pik To Cheung (Queen Mary Hospital, Hong Kong); Peter Colman (Walter & Eliza Hall Institute of Medical Research, Australia); Andrew Cotterill (Mater Children's Hospital, Australia); Jenny Couper (Women's and Children's Hospital, Australia); Patricia Crock (John Hunter Children's Hospital, Australia); Ric Cutfield (North Shore Hospital, New Zealand); Tim Davis (Fremantle Hospital, Australia); Paul Dixon (Diabetes Lifestyle Centre, New Zealand); Kim Donaghue (Children's Hospital at Westmead, Australia); Katrina Dowling (Australian Red Cross Blood Service, Australia); Paul Drury (Auckland Diabetes Centre, New Zealand); Sarah Dye (Western Australia Institute for Medical Research, Australia); Shane Gellert (The Royal Melbourne Hospital, Australia); Rohana Abdul Ghani (National University of Malaysia Hospital, Malaysia); Ristan Greer (University of Queensland, Australia); Xueyao Han (Peking University People's Hospital, China); Len Harrison (Walter & Eliza Hall Institute of Medical Research, Australia); Nick Homatopoulos (Australian Red Cross Blood Service, Australia); Linong Ji (Peking University People's Hospital, China); Tim Jones (Princess Margaret Hospital for Children, Australia); Loke Kah Yin (Children's Medical Institute, Singapore); Nor Azmi Kamaruddin (National University of Malaysia Hospital, Malaysia); Uma Kanga (All India Institute of Medical Sciences, India); Alok Kanungo (Cuttack Diabetes Research Foundation, India); Gurvinder Kaur (All India Institute of Medical Sciences, India); Betty Kek (Children's Medical Institute, Singapore); Simon Knowles (Australian Red Cross Blood Service, Australia); Jeremy Krebs (The Diabetes Centre, New Zealand); Neeraj Kumar (All India Institute of Medical Sciences, India); Yann---Jinn Lee (Mackay Memorial Hospital, Taiwan); Xiaoying Li (Shanghai Jiao---Tong University, China); Supawadee Liktimaskul (Mahidol University, Thailand); Margaret Lloyd (Children's Hospital at Westmead, Australia); Amanda Loth (Walter & Eliza

Hall Institute of Medical Research, Australia); Anthony Louey (Australian Red Cross Blood Service, Australia); Narinder Mehra (All India Institute of Medical Sciences, India); Tony Merriman (University of Otago, New Zealand); Liu Min (Beijing Children's Hospital, China); Grant Morahan (Western Australia Institute for Medical Research, Australia); Robert Moses (Illawarra Diabetes Services, Australia); Grant Mraz (Australian Red Cross Blood Service, Australia); Rinki Murphy (Auckland Diabetes Centre, New Zealand); Ian Nicholson (Australian Red Cross Blood Service, Australia); Araceli Panelo (Institute for Studies on Diabetes Foundation, Philippines); Perlita Poh (Royal Melbourne Hospital, Australia); Gareth Price (Mater Medical Research Institute, Australia); Nirubasini Ratnam (Princess Margaret Hospital for Children, Australia); Carani Sanjeevi (Karolinska Hospital, Sweden); Saikiran Sedimbi (Karolinska Hospital, Sweden); Shuixian Shen (Fudan University, China); Goh Siok Ying (The Children's Medical Institute, Singapore); Brian Tait (Australian Red Cross Blood Service, Australia); Nikhil Tandon (All India Institute of Medical Sciences, India); Allison Thomas (Walter & Eliza Hall Institute of Medical Research, Australia); Mike Varney, (Australian Red Cross Blood Service, Australia); Praewvarin Weerakulwattana (Mahidol University, Thailand); Jinny Willis (Christchurch Hospital, New Zealand).

European Network: Elvis Abang Akwo (Yaounde Central Hospital, Cameroon); Lotte Albret (Hagedorn Research Institute and Steno Diabetes Center, Denmark); Francisco Ampudia---Blasco (Clinic University Hospital Valencia, Spain); Jesus Argente (Hospital Infantil Universitario Nino Jesus, Spain); Magdalena Avbelj (University Children's Hospital, Slovenia); Gulja Babadjanova (Moscow State Medical University, Russia); Klaus Badenhoop (University Clinic Frankfurt/Main, Germany); Tadej Battelino (University Children's Hospital, Slovenia); Georg Beilhack (University of Ulm, Germany); Regine Bergholdt (Hagedorn Research Institute and Steno Diabetes Center, Denmark); Polly Bingley, (University of Bristol, United Kingdom); Bernhard Boehm (Ulm University, Germany); Jo Bolidson (University of Bristol, United Kingdom); Kerstin Brismar (Karolinska Hospital, Sweden); Caroline Brorsson (Hagedorn Research Institute and Steno Diabetes Center, Denmark); Joyce Carlson, (University Hospital MAS, Sweden); Luis Castano (Hospital de Cruces, Spain); Kyla Chandler (University of Bristol, United Kingdom); Valentino Cherubini (Salesi Hospital, Italy); Ondrej Cinek (Motol University Hospital, Czech Republic); Elisa

Cipponeri (University Campus Bio---Medico, Italy); Raquel Corripio Collado (Consorci Sanitari Parc Tauli, Spain); Alberto de Leiva (Hospital Sant Pau, Spain); Iveta Dzivite (University Children's Hospital, Latvia); Ana Fagulha (University Hospital, Portugal); Merce Fernandez Balcells (Hospital Trueta, Spain); Beatriz Garcia Cuartero (Hospital Severo Ochoa, Spain); Concepcion Garcia Lacalle (Hospital Severo Ochoa, Spain); Cristian Guja (Institute of Diabetes, Nutrition & Metabolic Diseases, Romania); Pilar Gutiérrez (Hospital Universitario de Getafe, Spain); Alona Hamou (Schneider Children's Medical Center of Israel, Israel); Erifili Hatziagelaki (University of Athens, Greece); Simon Heath (Centre National de Genotypage, France); Kaire Heilman (Tartu University Children's Hospital, Estonia); Wolfgang Helmberg, (Medical University Graz, Austria); Orna Hermon (Schneider Children's Medical Center of Israel, Israel); Marta Hernandez (Hospital Universitari Arnau de Vilanova and Hospital Universitario de Canarias, Spain); Iris Holzheu (Ulm University, Germany); Nora Hosszufalusi (Semmelweis University, Hungary); Jorma Ilonen (University of Turku, Finland); Constantin Ionescu---Tirgoviste (Institute of Diabetes, Romania); Jesper Johannesen (Steno Diabetes Center, Denmark); Cecile Julier (Centre National de Genotypage, France); Heinrich Kahles (Klinikum der J.W. Goethe---Universität, Germany); Ida Kinalska (Medical University of Bialystok, Poland); Mikael Knip (University of Helsinki, Finland); Ingrid Kockum, (Karolinska Hospital, Sweden); Eija Kojo (University of Helsinki, Finland); Olga Kordonouri (Children's Hospital auf der Bult, Germany); Adam Kretowski (Medical University of Bialystok, Poland); Dora Krikovszky (Semmelweis University, Hungary); Angelika Kurkhaus (Ulm University, Germany); Madiusz Kuzmicki (Medical University of Bialystok, Poland); Eva Lavant (University Hospital MAS, Sweden); Anna Long (University of Bristol, United Kingdom); Johnny Ludvigsson (University Hospital, Sweden); Laszlo Madacsy (Semmelweis University, Hungary); Katarzyna Maliszewska (Medical University of Bialystok, Poland); Mara Marga (P. Stradins University Hospital, Latvia); Marissa Penna Martinez (University Clinic Frankfurt/Main, Germany); Didac Mauricio (Hospital Universitari Arnau de Vilanova and Hospital Sant Pau, Spain); Gertrud Mazurkievicz (Ulm University, Germany); Jorn Nerup, (Steno Diabetes Center, Denmark); Antanas Norkus (Institute of Endocrinology of Kaunas University of Medicine, Lithuania); Francisco Javier Novoa Mogollon (Hospital Universitario Insular, Spain); Anna Okruszko (Medical University of Bialystok, Poland); Chiara Pettinari

(Salesi Hospital, Italy); Moshe Phillip (Schneider Children's Medical Center of Israel, Israel); Valdis Pirags (P. Stradins University Hospital, Latvia); Flemming Pociot (Hagedorn Research Institute and Steno Diabetes Center, Denmark); Paolo Pozzilli (University Campus Bio---Medico, Italy); Radu Racasan (University Clinic Frankfurt/Main, Germany); Klemens Raile (Vircow Clinic Charité Berlin, Germany); Rebecca Rappner (University Hospital MAS, Sweden); Maria Jesus Rodriguez Troyano (University Hospital of Las Palmas de Gran Canaria, Spain); Bart O. Roep (Leiden University Medical Center, Netherlands); Saba Rokni (Southmead Hospital, United Kingdom); Silke Rosinger (Ulm University, Germany); Oscar Rubio---Cabezas (Hospital Infantil Universitario Nino Jesus, Spain); Christa Ruckgaber (Ulm University, Germany); Ilhan Satman (Istanbul University, Turkey); Edith Schober (University Children's Hospital, Austria); Jochen Seufert (Medizinsche Poliklinik der Universität, Germany); Rosi Sing (Ulm University, Germany); Jan Skrha (Faculty of Medicine , Czech Republic); Eugene Sobngwi (Central National Obesity Centre and Hospital of Diabetes Endocrine, Cameroon); Michelle Somerville (University of Bristol, United Kingdom); Giatgen Spinas (University Hospital, Switzerland); Zdenek Sumnik (University Hospital Motol, Czech Republic); Vallo Tilmann (Tartu University Children's Hospital, Estonia); Dag Undlien (University of Oslo, Norway); Vaidotas Urbanavicius (Vilnius University Hospital, Lithuania); Bart Van der Auwera (Vrije Universiteit Brussel, Belgium); Federico Vasquez San Miguel (Hospital de Cruces, Spain); Andriani Vazeo---Gerasimidi (Diabetes Center P&A Kyriakou Children's Hospital, Greece); Dzilda Velickiene (Institute of Endocrinology of Kaunas University of Medicine, Lithuania); Ana Wägner, (University Hospital of Las Palmas de Gran Canaria, Spain, and Steno Diabetes Center, Denmark); Markus Walter (Diabetes Research Institute, Germany); Alistair Williams (University of Bristol, United Kingdom); Anette Ziegler (Diabetes Research Institute, Germany).

North American Network: Matthew Agleham (Roche Molecular Systems, United States); Alan Aldrich, (Benaroya Research Institute, United States); Ramin Alemzadeh (Medical College of Wisconsin, United States); Chester Alper (Immune Disease Institute, United States); Theresa Aly (Barbara Davis Center for Childhood Diabetes, United States); Dimitris Anastassiou (Columbia University, United States); Shaily Arora (Children's Hospital Oakland Research Institute, United States); Audrey Austin

(Children's National Medical Center, United States); Dorothy Becker (Rangos Research Center, United States); Christophe Benoist (Joslin Diabetes Center, United States); Noureddine Berka (Calgary Laboratory Services, Canada); Suruchi Bhatia (Oakland Children's Hospital Research Center, United States); Persia Bonella (Roche Molecular Systems, United States); Nunzio Bottini (University of Southern California, United States); Sean Boyle (Roche Molecular Systems, United States); Jeanah Braden (Children's Hospital Oakland Research Institute, United States); Barry Brady (Arkansas Children's Hospital, United States); Wendy Brickman (Children's Memorial Hospital, United States); Richard Christensen (Humphreys Diabetes Center, United States); Patrick Concannon (University of Virginia, United States); Robert Couch (University of Alberta, Canada); Debra Counts (University of Maryland, United States); Jill Crandall (Albert Einstein College of Medicine, United States); Mark Daniels (Children's Hospital of Orange County, United States; Larry Dolan (Cincinnati Children's Hospital Medical Center, United States); David Donaldson (Utah Diabetes Center, United States); Alessandro Doria (Joslin Diabetes Center, United States); George Eisenbarth (Barbara Davis Center for Childhood Diabetes, United States); James Elder (University of Michigan, United States); Rita El---Hajj (Main Line Health Heart Center, United States); Henry Erlich (Roche Molecular Systems, United States); Pamela Fain (Barbara Davis Center for Childhood Diabetes, United States); Anna Lisa Fear (Children's Hospital Oakland Research Institute, United States); Robert Ferry (The University of Texas Health Science Center at San Antonio, United States); Rosanna Fiallo---Scharer (Barbara Davis Center for Childhood Diabetes, United States); Daniel Geraghty (Fred Hutchinson Cancer Research Center, United States); Soumitra Ghosh (Medical College of Wisconsin, United States); Steven Gitelman (University of California at San Francisco, United States); Michelle Godwin (Fred Hutchinson Cancer Research Center, United States); Robin Goland (Naomi Berrie Diabetes Center, United States); Nathan Goodman (Institute for Systems Biology, United States); Greg Goodwin (Joslin Diabetes Center, United States); Jenna Gravely (Fred Hutchinson Cancer Research Center, United States); Carla Greenbaum (Benaroya Research Institute, United States); Chelsea Gudgeon (Fred Hutchinson Cancer Research Center, United States); Fred Gunville (Billings Clinic, United States); William Hagopian (University of Washington, United States); Hakon Hakonarson (Children's Hospital of Philadelphia, United States); John Hansen, (Fred Hutchinson Cancer Research Center, United States); Kimberly Harrington (Fred Hutchinson Cancer Research Center, United States); Jeanne Hassing

(Sacred Heart, United States); Wendy Hilliker (Fred Hutchinson Cancer Research Center, United States); Robert Hoffman (Ohio State University, United States); Erin Hulbert (Institute for Systems Biology, United States); Roberto Izquierdo (SUNY Upstate Medical University, United States); Nicholas Jospe (University of Rochester, United States); Kevin Kaiserman (Children's Hospital Los Angeles, United States); Francine Kaufman (Children's Hospital Los Angeles, United States); Samuel Kim (Roche Molecular Systems, United States); Erin Kloos (Fred Hutchinson Cancer Research Center, United States); Roman Kosoy (Benaroya Research Institute, United States); James Lane (University of Nebraska, United States); Julie Lane (Children's Hospital Oakland Research Institute, United States); Jean Lawrence (Kaiser Permanente, United States); Claresa Levetan (Main Line Health Heart Center, United States); Phil Levin (MODEL Clinical Research, United States); Rebecca Lipton (University of Chicago, United States); John Lonsdale (Human Biological Data Interchange, United States); Victoria Magnuson (Children's Hospital of Wisconsin, United States); Jennifer Marks (University of Miami, United States); Beth Mayer--Davis (University of South Carolina, United States); Robert McEvoy (Children's Hospital of Minnesota, United States); Richard McIndoe (Medical College of Georgia, United States); Lesley Merkle (Fred Hutchinson Cancer Research Center, United States); Daniel Metzger (BC Children's Hospital, Canada); Dongmei Miao (Barbara Davis Center for Childhood Diabetes, United States); Eric Mickelson (Fred Hutchinson Cancer Research Center, United States); Priscilla Moonsamy (Roche Molecular Systems, United States); Wayne Moore (Children's Mercy Hospital, United States); Antoinette Moran (University of Minnesota, United States); Janelle Noble (Children's Hospital Oakland Research Institute, United States); Gary Olsem (Fred Hutchinson Cancer Research Center, United States); Suna Onengut---Gumuscu (University of Virginia, United States); Tihamer Orban (Joslin Diabetes Center, United States); Craig Orlowski (University of Rochester Medical Center, United States); Andrew Paterson (University of Toronto, Canada); Massimo Pietropaolo (University of Michigan Medical School, United States); Catherine Pihoker (Children's Hospital and Regional Medical Center, United States); Constantin Polychronakos, (McGill University Health Center, Canada); Jeff Post (Roche Molecular Systems, United States); Daniel Postellon (Helen DeVos Children's Hospital, United States); Alberto Pugliese, (University of Miami, United States); HuiQi Qu (Montreal Children's Hospital, Canada); Teresa Quattrin (Women and Children's Hospital of Buffalo, United States); Mark Rappaport

(Pediatric Endocrine Associates, United States); Philip Raskin (University of Texas Southwestern Medical Center, United States); Heather Risbeck (Fred Hutchinson Cancer Research Center, United States); Henry Rodriguez (Riley Hospital for Children, United States); Luisa Rodriguez (Baylor College of Medicine, United States); Michelle Rogers (Fred Hutchinson Cancer Research Center, United States); Leticia Rubalcava (Children's Hospital Oakland Research Institute, United States); Bill Russell (Vanderbilt University, United States); Desmond Schatz (University of Florida, United States); Carla Scott (University of Texas Health Science Center at San Antonio, United States); Jin---Xiong She (Medical College of Georgia, United States); Heather Shilling (Benaroya Research Institute, United States); Dorothy Shulman (University of South Florida, United States); Leslie Soyka (University of Massachusetts Memorial Center, United States); Phyllis Speiser (Schneider Children's Hospital, United States); Harold Starkman (Atlantic Health System, United States); Andrea Steck (Barbara Davis Center for Childhood Diabetes, United States); Sarah Stender (University of Tennessee, United States); Lorraine Stratton (University of Arizona, United States); Daniel Sur (Roche Molecular Systems, United States); Shayne Taback (University of Manitoba, United States); Kathryn Thrailkill (Arkansas Children's Hospital, United States); Ellen Toth (University of Alberta, Canada); Patricia Trymbiski (Doylestown Hospital, United States); Eva Tsalikian (University of Iowa, United States); Katherine Vertachnik (Fred Hutchinson Cancer Research Center, United States); Jack Wahlen (Endocrine Research Specialists, United States); Xujing Wang (Max McGee National Research Center of Juvenile Diabetes, United States); Sandra Weber (Greenville Hospital System, United States); Diane Wherrett (Hospital for Sick Children, Canada); Steven Willi (Children's Hospital of Phildelphia, United States); Darrell Wilson (Stanford University, United States); Jerry Youkey (Greenville Hospital System, United States); Neal Young (National Institutes of Health, United States); Liping Yu (Barbara Davis Center for Childhood Diabetes, United States); Lue Ping Zhao (Fred Hutchinson Cancer Research Institute, United States); Donald Zimmerman (Children's Memorial Hospital, United States).

United Kingdom Network: Ellen Adlem (University of Cambridge, United Kingdom); James Allen (University of Cambridge, United Kingdom); Jeffrey Barrett (WT Sanger Institute, United Kingdom); Judy Brown (University of Cambridge, United Kingdom); Oliver Burren (University of Cambridge, United Kingdom); Pamela Clarke (University

of Cambridge, United Kingdom); David Clayton (University of Cambridge, United Kingdom); Gillian Coleman (University of Cambridge, United Kingdom); Jason Cooper (University of Cambridge, United Kingdom); Francesco Cucca (University of Sassari, United Kingdom); Lucy Davison (University of Cambridge, United Kingdom); Kate Downes (University of Cambridge, United Kingdom); Simon Duley (University of Cambridge, United Kingdom); David Dunger (University of Cambridge, United Kingdom); Laura Esposito (University of Cambridge, United Kingdom); Vin Everett (University of Cambridge, United Kingdom); Sarah Field (University of Cambridge, United Kingdom); Jason Hafler (University of Cambridge, United Kingdom); Matthew Hardy (University of Cambridge, United Kingdom); Deborah Harrison (University of Cambridge, United Kingdom); Inge Harrison (University of Cambridge, United Kingdom); Steve Hawkins (University of Cambridge, United Kingdom); Barry Healy (University of Cambridge, United Kingdom); Simon Hood (University of Cambridge, United Kingdom); Simon Howell (King's College, United Kingdom); Joanna Howson (University of Cambridge, United Kingdom); Meeta Maisuria (University of Cambridge, United Kingdom); William Meadows (University of Cambridge, United Kingdom); Trupti Mistry (University of Cambridge, United Kingdom); Sergey Nezhenstsev (University of Cambridge, United Kingdom); Sarah Nutland, (University of Cambridge, United Kingdom); Nigel Ovington (University of Cambridge, United Kingdom); Vincent Plagnol (University of Cambridge, United Kingdom); Dan Rainbow (University of Cambridge, United Kingdom); Kara Rainbow (University of Cambridge, United Kingdom); Srilakshmi Raj (University of Cambridge, United Kingdom); Helen Schuilenburg (University of Cambridge, United Kingdom); Anna Simpson (University of Cambridge, United Kingdom); Luc Smink (University of Cambridge, United Kingdom); Debbie Smyth (University of Cambridge, United Kingdom); Helen Stevens (University of Cambridge, United Kingdom); Niall Taylor (University of Cambridge, United Kingdom); John Todd (University of Cambridge, United Kingdom); Jaakko Tuomilehto (National Public Health Institute, Finland); Neil Walker, (University of Cambridge, United Kingdom); Linda Wicker (University of Cambridge, United Kingdom); Barry Widmer (University of Cambridge, United Kingdom); Mark Wilson (University of Cambridge, United Kingdom); Heather Withers, (University of Cambridge, United Kingdom); Jennie Yang (University of Cambridge, United Kingdom).

Coordinating Center: Mark Brown (Wake Forest University Health Sciences, United States); Wei---Min Chen (University of Virginia, United States); Arnetta Crews (Wake Forest University Health Sciences, United States); Jason Griffin (Wake Forest University Health Sciences, United States); Mark Hall (Wake Forest University Health Sciences, United States); Teresa Harnish (Wake Forest University Health Sciences, United States); John Hepler (Wake Forest University Health Sciences, United States); Joan Hilner (Wake Forest University Health Sciences, United States); Nancy King (Wake Forest University Health Sciences, United States); Kurt Lohman (Wake Forest University Health Sciences, United States); Lingyi Lu (Wake Forest University Health Sciences, United States); Josyf Mychaleckyj, (University of Virginia, United States); Jay Nail (Wake Forest University Health Sciences, United States); Letitia Perdue, (Wake Forest University Health Sciences, United States); June Pierce (Wake Forest University Health Sciences, United States); David Reboussin, (Wake Forest University Health Sciences, United States); Stephen Rich (University of Virginia, United States); Scott Rushing (Wake Forest University Health Sciences, United States); Michele Sale (University of Virginia, United States); Elizabeth Sides, (Wake Forest University Health Sciences, United States); Beverly Snively (Wake Forest University Health Sciences, United States); Hoa Teuschler (Wake Forest University Health Sciences, United States); Goodrich Theil (Wake Forest University Health Sciences, United States); Lynne Wagenknecht (Wake Forest University Health Sciences, United States); Dustin Williams (Wake Forest University Health Sciences, United States).

Project Office: Beena Akolkar (National Institute of Diabetes and Digestive and Kidney Diseases/National Institutes of Health, United States); Catherine McKeon (National Institute of Diabetes and Digestive and Kidney Diseases/National Institutes of Health, United States); Concepcion Nierras (Juvenile Diabetes Research Foundation International, United States); Elizabeth Thomson (National Human Genome Research Institute/National Institutes of Health, United States).

Other Contributors: David Altshuler (Whitehead Institute for Biomed Research, United States); Kinman Au (Medical College of Georgia, United States); Steve Bain (University of Wales Swansea, United Kingdom); Lisa Barcellos (University of California at Berkeley, United States); Sandra Barral (Rockefeller University, United States); Tim Becker (Karolinska Institutet, Sweden); Farren Briggs (University of

California at Berkeley, United States); Paola Bronson (University of California at Berkeley, United States); Mark Daly, (Massachusetts General Hospital, United States); Paul de Bakker (Massachusetts General Hospital, United States); Panos Deloukas (Wellcome Trust Sanger Institute, United Kingdom); Bernie Devlin (University of Pittsburgh, United States); Morten Chrisoph Eike, (Institute of Immunology, Norway); Leigh Field (University of British Columbia, Canada); Stacey Gabriel (Broad Institute of MIT and Harvard, United States); Nikhil Garge (Medical College of Georgia, United States); Silvana Gaudieri (Murdoch University, Australia); Ben Goldstein (University of California at Berkeley, United States); Clara Gorodezky (INDRE SSA, Mexico); Sara Hamon (Rockefeller University, United States); Chungsheng He (Rockefeller University, United States); Joanna Howson (University of Cambridge, United Kingdom); Keith Humphreys (Karolinska Institutet, Sweden); Ian James (Murdoch University, Australia); Mark Lathrop (Centre National de Genotypage, France); Benedicte Alexandra Lie (University of Oslo, Norway); Dawei Li (Rockefeller University, United States); Steven Mack (Roche Molecular Systems, United States); Ralph McGinnis (Wellcome Trust Sanger Institute, United Kingdom); Elizabeth McKinnon (Murdoch University, Australia); William McLaren (Wellcome Trust Sanger Institute, United Kindgom); David Nolan (Murdoch University, Australia); Marita Olsson (Karolinska Institutet, Sweden); Jurg Ott (Rockefeller University, United States); David Owerbach (Baylor College of Medicine, United States); Chris Patterson (Queen's University Belfast, United Kingdom); Robert Podolsky (Medical College of Georgia, United States); Patricia Ramsay (University of California at Berkeley, United States); Venkatesh Rangantah (Wellcome Trust Sanger Institute, United Kingdom); Neil Risch (University of California at San Francisco, United States); Kjersti Skjold Ronningen (Norwegian Institute of Public Health, Norway); Xiarong Shao (University of California at Berkeley, United States); Richard Single (University of Vermont, United States); Michael Steffes (University of Minnesota, United States); Glenys Thomson (University of California at Berkeley, United States); Ana Maria Valdes, (Lartech, Italy); Claire Vandiedonck (Wellcome Trust Centre for Human Genetics, United Kingdom); Pam Whittaker (Wellcome Trust Sanger Institute, United Kindgom); Qingrun Zhang (Beijing Institute of Genomics, China).
